# Supplementary material for: The role of natural gas in reaching net-zero emissions in the electric sector
Source: Nat Commun. 2022 Aug 12;13:4743. doi: 10.1038/s41467-022-32468-w (PMC9374715; doi:10.1038/s41467-022-32468-w)
Supplement: Supplementary file 1 — Supplementary Information [file 41467_2022_32468_MOESM1_ESM.pdf]

Supplementary Information

The Role of Natural Gas in Reaching Net-Zero Emissions in the Electric Sector

John E.T. Bistline, David T. Young

Supplementary Note 1: Overview of Methods

EPRI's U.S. Regional Economy, Greenhouse Gas, and Energy (REGEN) model finds intertemporally cost-optimal electric sector pathways that meet demand given assumptions about policies, technologies, and markets. It represents 16 distinct regions of the continental U.S. interconnected by transmission and trade (Supplementary Figure 1). For each time period, the model determines generation capacity investments, generation dispatch, storage charge/discharge, hydrogen production/storage, transmission and CO_2 pipeline investments, and other parameters to minimize the net present value of the electric sector costs to meet demand in every hour subject to capacity, policy, and technology constraints.

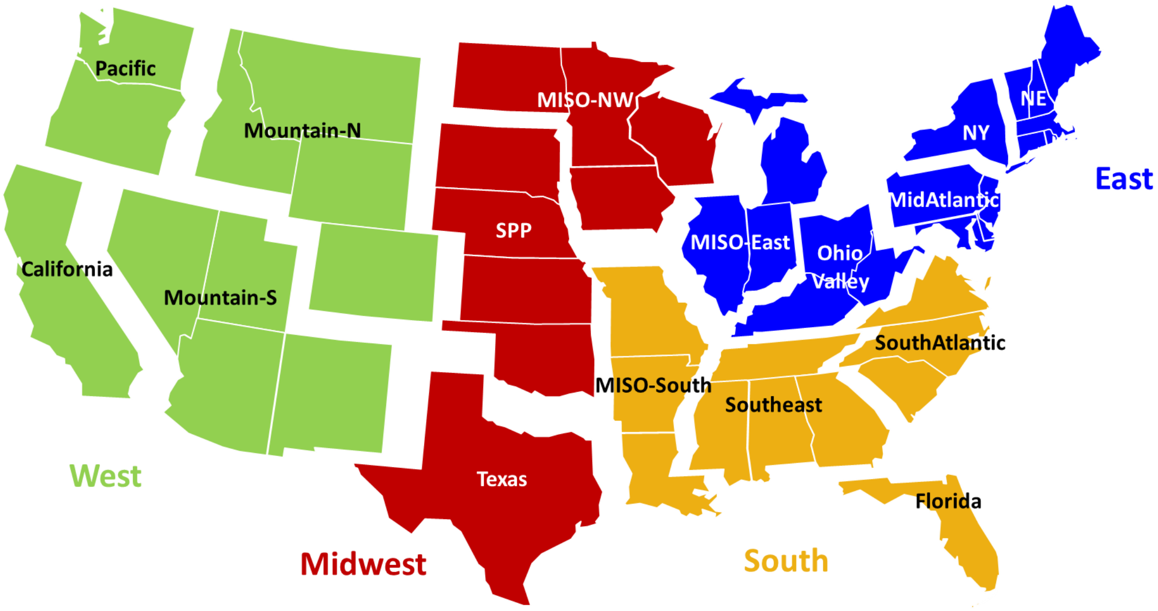

Supplementary Figure 1. Regional aggregation of the REGEN model for this analysis. Four reporting regions are also shown.

The electric sector capacity expansion model is integrated with a technologically detailed consumer choice model of end-use service and energy demand, with the same geographic disaggregation and hourly temporal resolution. The two models are run iteratively, with the electric model passing hourly electricity prices to the end-use model, and the end-use model passing back hourly load shapes and load growth, until energy prices and demands converge between the two models (Supplementary Figure 2).

The end-use model includes structural detail across several dimensions relevant for fuel and technology choice, such as building size, type, and vintage, climate zone and location, and vehicle ownership and driving intensity. Within each structural category, service demand may be met with a range of options,

characterized as combinations of fuels and technologies. The model evaluates the total cost of each option in each new vintage based on assumed technology cost and performance, fuel prices, structural attributes of service demand, and non-economic factors. The resulting allocation across the options is based on a logit model translating relative costs to equilibrium market shares, with a lagged process to simulate a gradual transition toward the model's calculated equilibrium shares. The model then calculates annual and hourly fuel use by region as a function of the resulting mix of end-use technologies. Investment costs for end-use technologies (e.g., light-duty electric vehicles, air-source heat-pumps) are sourced from the literature and EPRI expert elicitations. Fuel costs are sourced from the U.S. EIA *Annual Energy Outlook* reference case [1], which also serve as inputs to the electric model. The cost of electricity is an input provided by the electric sector model.

The end-use model includes a separate module to evaluate rooftop solar PV adoption, investments in which are considered from the perspective of retail consumers, where installing behind-the-meter generation may offset retail purchases at flat volumetric rates. Rates are calculated using price outputs from the electric model and assume today's existing rate structures. The US-REGEN documentation Section 3.6 has more information [2].

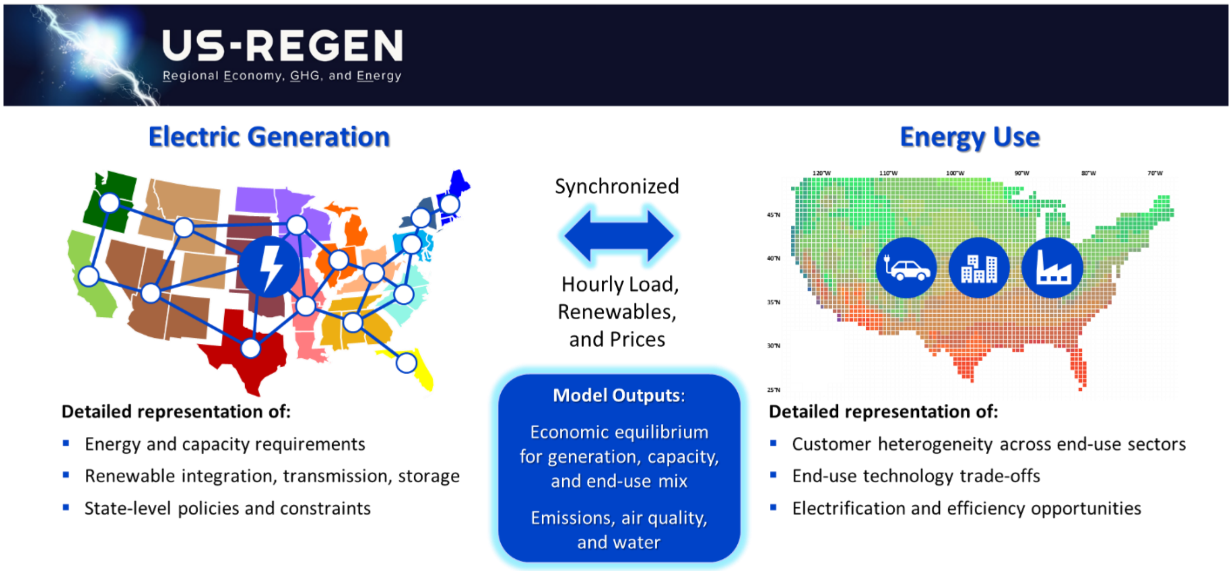

Supplementary Figure 2. Overview of the US-REGEN model. Full model documentation and other recent reports can be found at https://esca.epri.com/usregen.

Electric vehicle charging shapes vary by household type, charging location, and day of the week and reflect a mix of charging locations (home, work, public) and power levels [3]. Note that these shapes represent aggregate charging patterns rather than individual vehicle shapes. These charging profiles are based on research by EPRI's Electric Transportation program and come from a combination of aggregated empirical data and simulation. US-REGEN also provides options for flexible charging, including for a fleet of autonomous vehicles. The participation share in charging flexibility programs is assumed to be 50% for residential households and 80% for workplace charging. The flexible charging module in US-REGEN minimizes the cost of charging subject to constraints that the daily vehicle energy requirement for each profile must be met in the available hours and that charging in each hour is limited

by available capacity. The focus of the charging module is load shifting, where vehicle charging shifts in time as response to utility control, time-varying pricing, or other incentives.

Hourly regional renewable output and resource potentials are based on analysis and data by EPRI, AWS Truepower, and NASA's MERRA-2 dataset and provide synchronous time-series values with load. Variability is modeled using gridded hourly data from NASA's MERRA-2 dataset, which provides key meteorological variables such as wind speed, solar irradiation, and temperature. For wind technologies, wind speed at hub height (which ranges from 80 to 140 meters) is translated into power output based on assumed power curves for a range of turbine technologies. Wind output profiles for given regions and resource classes vary by vintage based on an assumed mix of turbine type and hub height, as detailed in EPRI (2020) [2]. Solar output profiles are derived from gridded hourly radiation flux data from MERRA-2. Diffuse and direct irradiance are translated into output for a variety of solar photovoltaic technologies that are specified in terms of the orientation and tilt of the panels. Captured energy at the panel is adjusted for temperature impacts on module efficiency, non-linear inverter losses, and a gross de-rating factor reflecting a range of factors not otherwise captured. Additional detail is provided in Section 2.4.1 of the US-REGEN documentation [2].

Hourly profiles used in the model solution are based on a single representative year (2015 for these experiments), and the same underlying meteorology and temperatures are used in the end-use model to develop hourly load shapes (e.g., for electric space heating in residential and commercial buildings) to avoid dampening variance through multi-year averaging. While consideration of multiple weather years may reveal more extreme events, there are significant wind droughts observed in the sample year, reinforcing the importance of energy storage and firm resources for balancing. Moreover, the model includes a reserve requirement that firm capacity exceed the residual peak load in each region, suggesting the results are relatively robust to extended wind and solar droughts. Additional detail on wind and solar resource assumptions and technology characteristics is provided in Section 2.4 of the US-REGEN documentation [2].

The US-REGEN electric-sector model is run in two modes. In the dynamic mode, the model solves the inter-temporal capacity planning optimization problem across the years 2020–2050 in five-year periods but, due to computational constraints, uses 120 representative hours for each year. This mode cannot capture the hourly operations of short-duration energy storage technologies (e.g., lithium-ion battery storage), though longer-duration options are included. In the static mode, a shorter foresight optimization is cast as a single year static equilibrium model with capacity investment and hourly dispatch using the retirement information from the intertemporal solve. The use of static mode allows the analysis to represent hourly operations and capacity investments faithfully, something that is not currently possible in large-scale intertemporal models [4]. The literature has demonstrated that a model must be able to capture the declining economic value of variable renewable energy at higher penetration levels and ability of system resources like energy storage to mitigate these effects, which are captured in US-REGEN [2, 5]. This formulation is conceptually similar to a sequential myopic model [6].

Supplementary Note 2: Assumptions

Comparisons of historical electric sector CO_2 emissions and reductions under the policy scenarios are shown in Supplementary Figure 3. Non-electric sectors are assumed to have a carbon price as a proxy for decarbonization incentives at the end-use level, which is assumed to start at \$50/t-CO_2 in 2025 and increases at 7% per year.

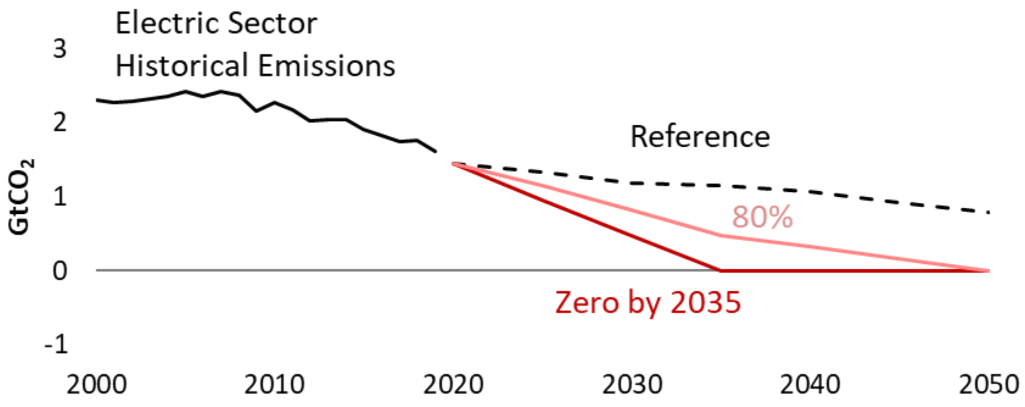

Supplementary Figure 3. Electric sector CO_2 emissions. Historical emissions are shown alongside model projections in the reference scenario and zero emissions scenarios by 2035 and 2050.

Cost and performance assumptions for other technologies come from the literature and EPRI's Technology Assessment Guide, for which a high-level summary is publicly available via EPRI's Integrated Technology Generation Options report [7]. These capital costs are shown alongside the lower cost optimistic sensitivity for variable renewables and batteries in Supplementary Figure 4.

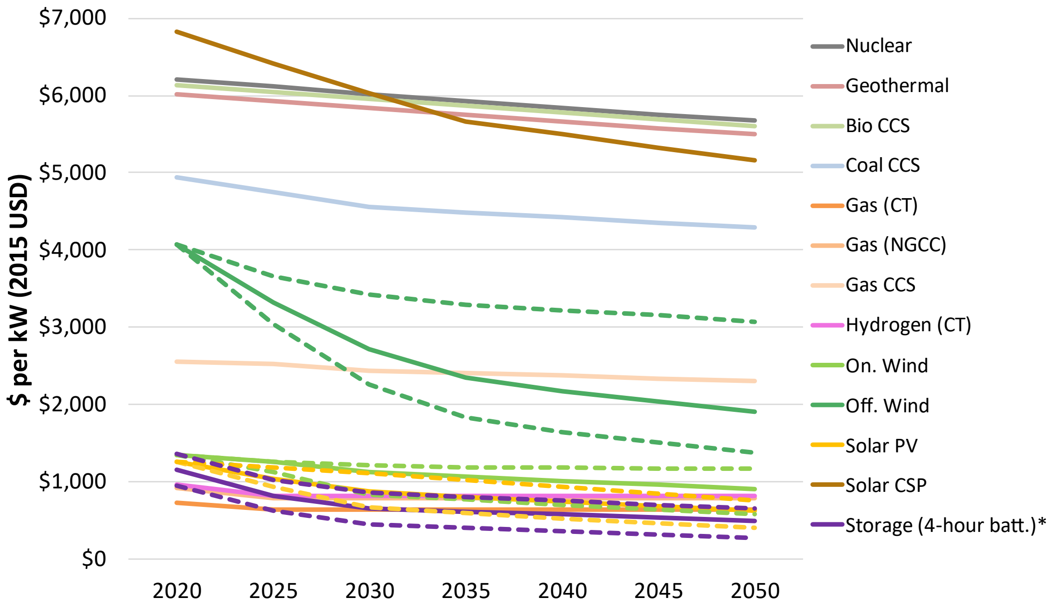

Supplementary Figure 4. Capital costs by technology over time. U.S. average costs are shown; modeled costs vary by state. High and Low renewables costs are shown as dotted lines. Energy storage costs are shown for four-hour battery storage; duration is endogenous in the model.

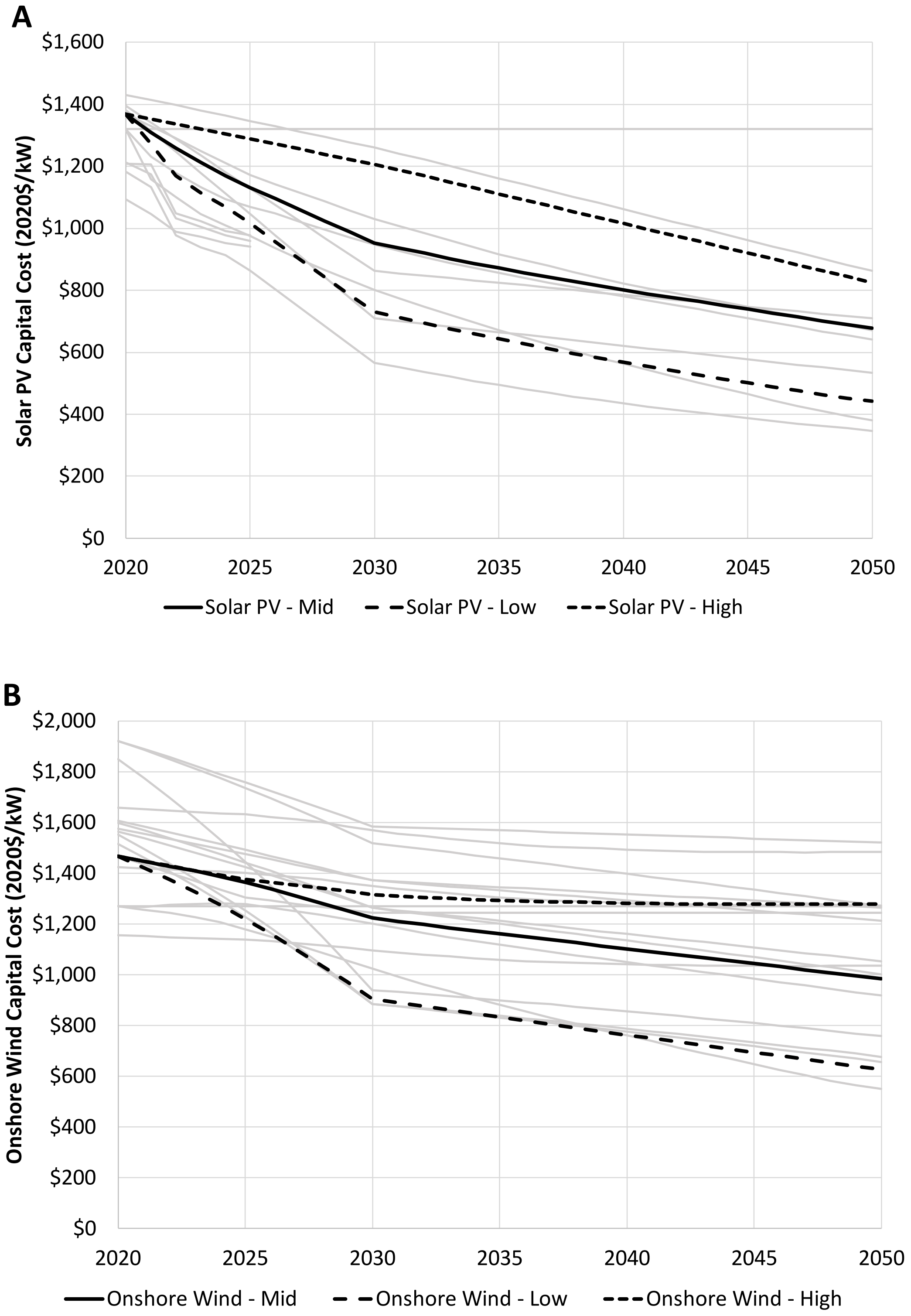

Supplementary Figure 5. Renewable cost projections over time across different scenarios and organizations. Light gray lines show U.S. projections from other organizations, and black lines show the

Low (dashed), Mid (solid), and High (dotted) sensitivities used in this analysis. (A) Solar PV with single-axis tracking in $/kW$_{AC}$ terms. (B) Onshore wind. All values are expressed in 2020 U.S. dollars.

Supplementary Figure 5 shows capital costs over time for single-axis tracking solar PV and onshore wind, comparing Low, Mid, and High scenarios from this analysis with several U.S. studies published in 2020 and 2021: U.S. Energy Information Administration's *Annual Energy Outlook* (Feb. 2021), National Renewable Energy Laboratory's *Annual Technology Baseline 2020* (Jul. 2020), BloombergNEF's *2H 2020 LCOE Data Viewer* (Jan. 2021), and Lawrence Berkeley National Laboratory's *Expert Predictions about the Future of Onshore and Offshore Wind Energy* (Apr. 2021). We include low, middle, and high cases from these studies and adjust to constant 2020 USD.

US-REGEN represents a range of energy storage technologies such as batteries, compressed air energy storage, existing pumped hydro, and hydrogen via electrolysis. For batteries, the model endogenously selects battery storage investment and system configurations (i.e., ratio of energy capacity to power capacity) based on cost structure assumptions from Minear, et al. (2020) [8]. The cost structure is specified in terms of the power rating/capacity costs ($/kW) and energy capacity costs ($/kWh). Supplementary Figure 6 illustrates cost reductions for four-hour lithium-ion systems over time for various cost scenarios. There are rapid cost declines through 2030 and slower post-2030 changes, which is consistent with other studies in the literature.

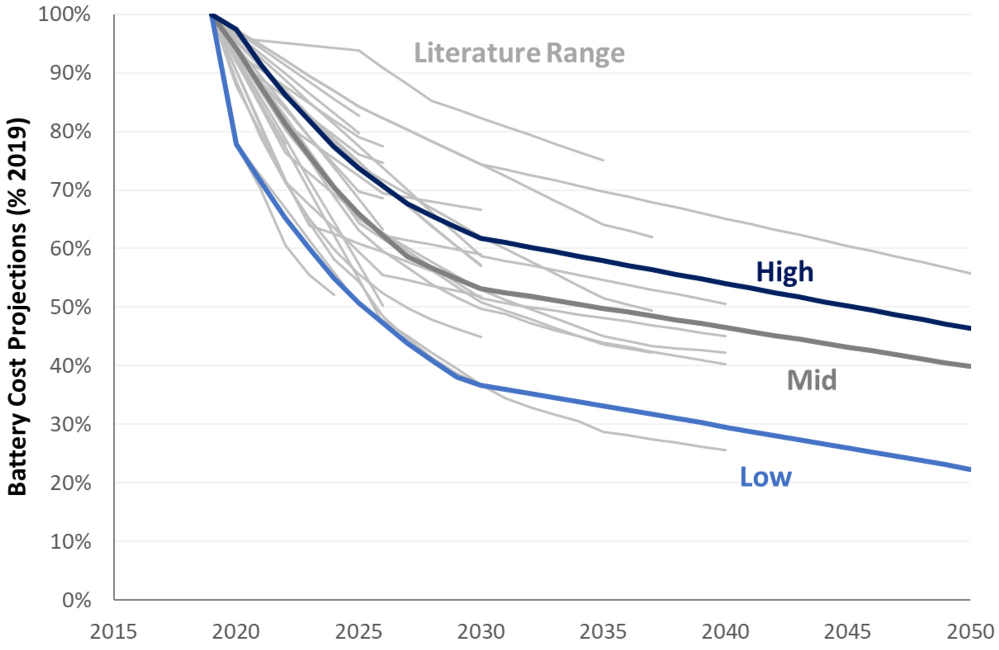

Supplementary Figure 6. Battery costs over time for low, mid, and high scenarios with literature projections. Projections for four-hour lithium-ion systems. Note that the duration of battery storage systems is endogenous in the model. The literature range comes from Cole and Frazier (2020), "Cost Projections for Utility-Scale Battery Storage: 2020 Update."

US-REGEN optimizes the capacity of hydrogen production via electrolysis and generation from hydrogen turbines. Electrolysis system capital costs are shown in Supplementary Figure **7**. Compared with estimates in the literature, US-REGEN costs are on the lower end of the range. The cost of electricity is

endogenously determined from the scenario-specific resource mix in the model. As described in detail in Section 2.4 of the full US-REGEN documentation [2], the model includes several other hydrogen production pathways, including steam methane reforming using natural gas (including options with and without CCS) and coal gasification (with and without CCS).

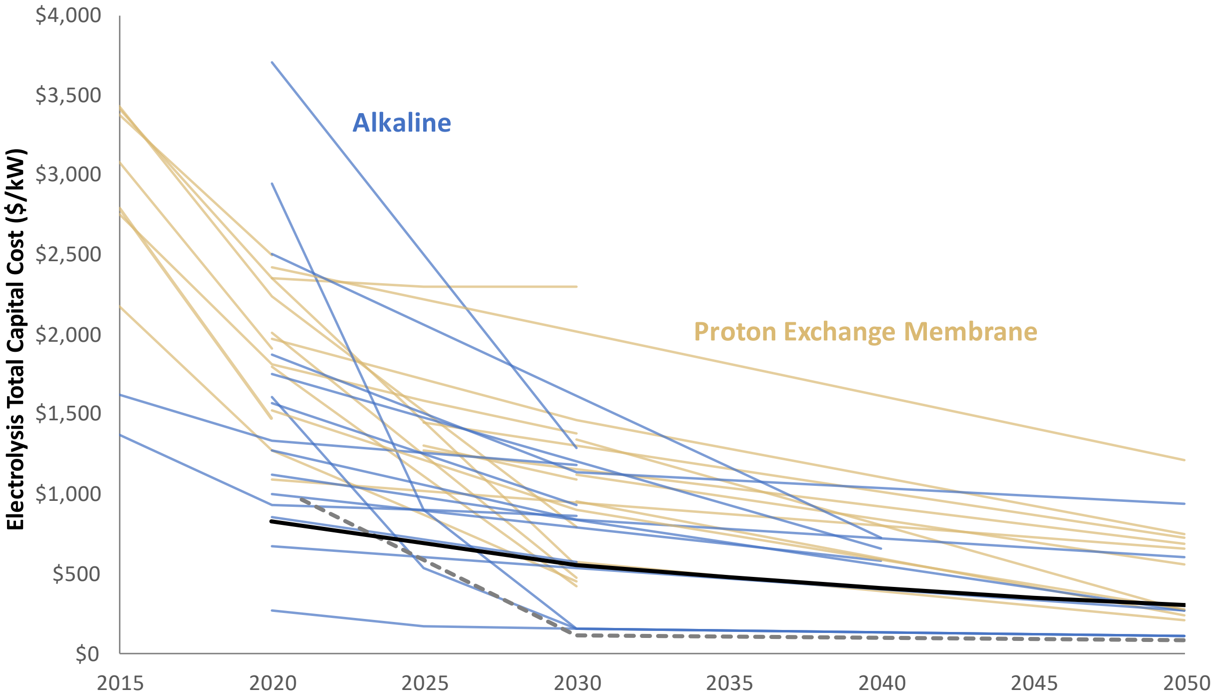

Supplementary Figure 7. Electrolysis system capital costs alkaline (blue) and proton exchange membrane (gold) technologies in the current literature. Cost projections come from a range of journal articles and reports summarized in [9]. US-REGEN reference costs (black line) and low costs (dotted line) are shown.

In addition to the BECCS costs shown in Supplementary Figure 4, CDR is also included in US-REGEN through several DAC configurations. DAC technologies vary by process—either high-temperature (HT) liquid solvent or low-temperature (LT) solid sorbent designs—and by heat supply—natural gas, electric, or hybrid. Supplementary Table 1 shows 2020 DAC cost and performance assumptions, which are expressed in terms of net CO_2 removed (i.e., including capture from flue gas when heat is provided by natural gas combustion). Capital costs for DAC are assumed to decline over time, per Supplementary Figure 8, where lower-temperature solid sorbent systems have larger percentage cost reductions from modularity but are still higher cost than high-temperature solvent designs across the modeling horizon.

Supplementary Table 1. DAC configurations and parameters assumptions in 2020. DAC technologies vary by process—high-temperature (HT) liquid solvent or low-temperature (LT) solid sorbent designs—and heat supply. All values are normalized to net removal at the plant per metric ton of CO_2. Assumptions are based on Keith, et al. (2018) [10] and NAS (2019) [11].

Parameter	HT-All Gas	HT-Hybrid	HT-All Electric	LT-Hybrid	LT-All Electric
**Capital Cost ($/t-$CO_2$/yr)**	$1,095	$653	$1,014	$1,765	$1,765

**Electricity Input (MWh/t-CO_2)**	0	0.366	3.711	0.232	0.232
**Gas Input (MMBtu/t-CO_2)**	8.35	4.976	0	3.886	0
**Variable O&M ($/t-$CO_2$)**	$11.30	$9.20	$9.70	$13.30	$13.30
**Fixed O&M ($/t-$CO_2$/yr)**	$90.10	$54.30	$86.50	$151.10	$151.10

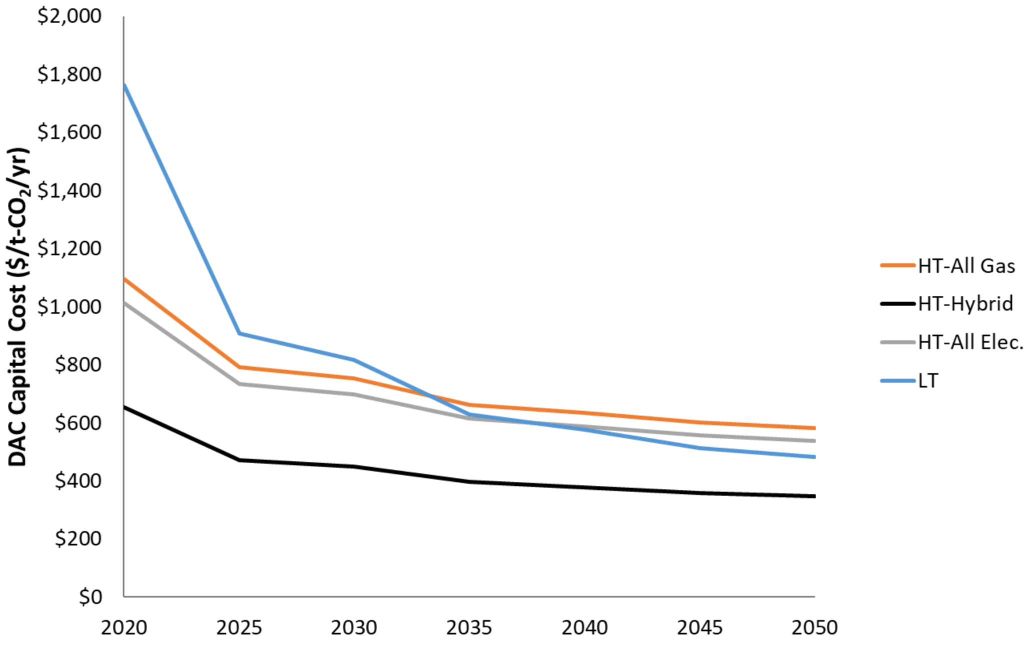

Supplementary Figure 8. DAC capital costs over time by technology. Assumptions are based on Keith, et al. (2018) [10], NAS (2019) [11], and Fasihi, et al. (2019) [12].

The model represents the transport of captured CO_2 to injection sites for storage in saline aquifers. There is regional heterogeneity in CO_2 transport and storage costs (Supplementary Figure 9), which varies based on the volume stored. State-specific CO_2 storage capacity is limited by values from the National Carbon Sequestration Database (NATCARB). Capital costs of CCS-equipped technologies include the cost of a 20-mile CO_2 pipeline that enables access to a dedicated injection site or large pipeline for interregional transport (Supplementary Figure 4). We assume that all captured CO_2 is permanently sequestered [13].

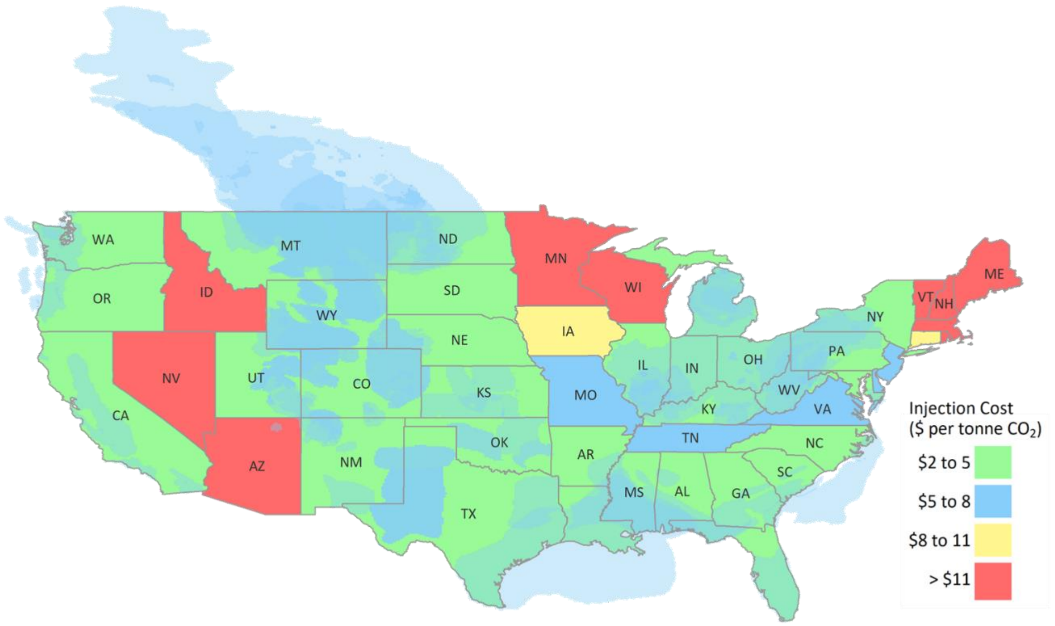

Supplementary Figure 9. CO_2 transport and storage costs by U.S. state. Costs include inter-regional transport for locations without storage capacity. Saline aquifers are shown in the blue shading. This figure is based on the US-REGEN documentation [2], which has additional information about the representation and assumptions for CO_2 transport and storage.

Regional biomass supply curves are shown in Supplementary Figure 10. These curves are based on the Forest and Agriculture Sector Optimization Model with Greenhouse Gases (FASOM-GHG). More information on the forestry and agricultural biomass supply modeling is provided in Appendix B of the US-REGEN documentation [2].

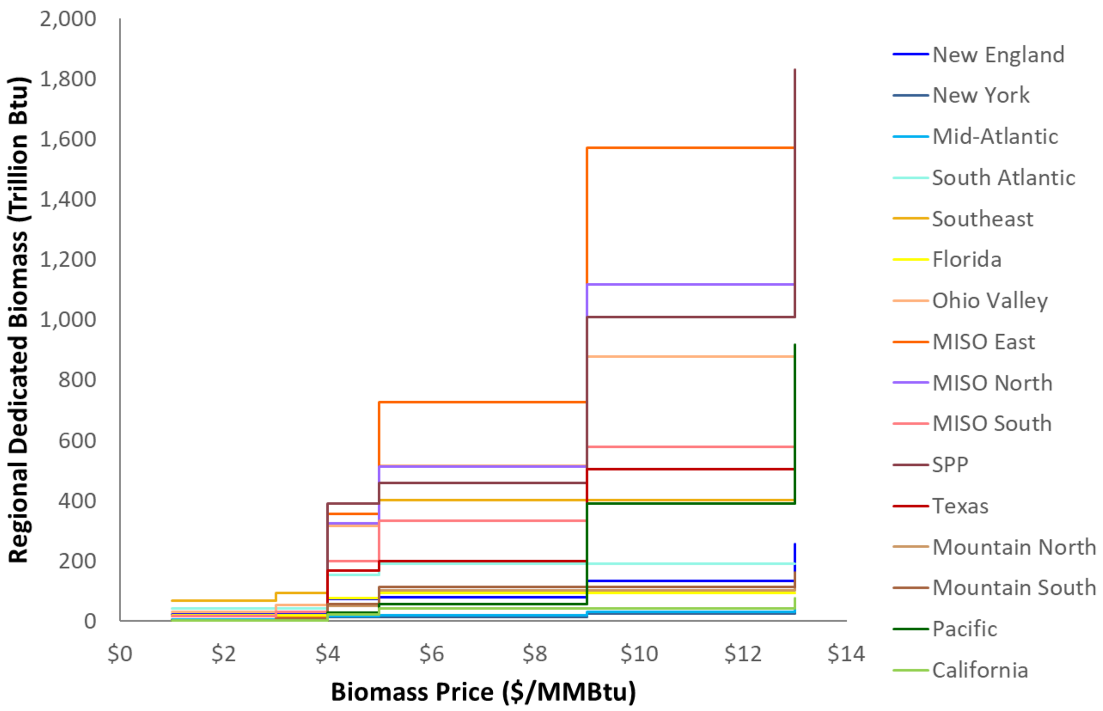

Supplementary Figure 10. Regional power sector biomass supply curves by US-REGEN model region in 2050. Delivered biomass costs for electricity production are based on the Forest and Agriculture Sector Optimization Model with Greenhouse Gases (FASOM-GHG).

Natural gas price assumptions are obtained from the U.S. Energy Information Administration's *Annual Energy Outlook 2020* "Reference" case [1], as shown in Supplementary Figure 11. Low and high gas price sensitivities come from the *Annual Energy Outlook 2020* "High Oil and Gas Supply" and "Low Oil and Gas Supply" scenarios, respectively.

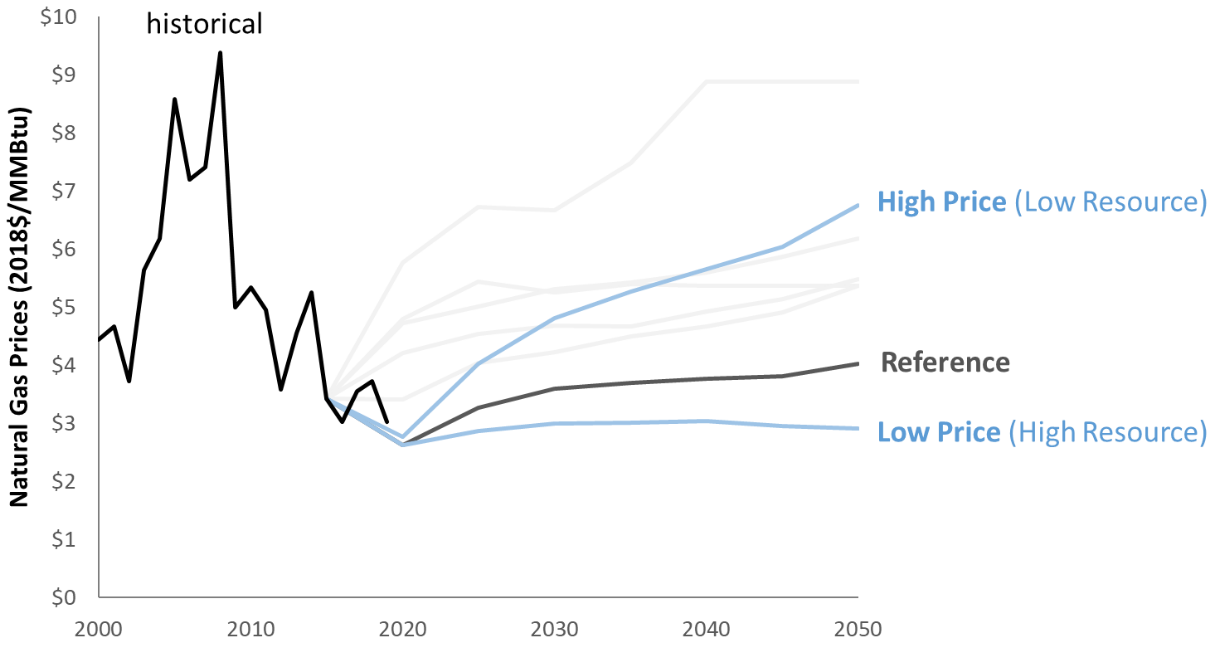

Supplementary Figure 11. Natural gas prices over time, including historical prices and projections. Projections are for delivered prices to the electric sector and come from the U.S. Energy Information Administration's *Annual Energy Outlook*.

Supplementary Table 2 provides detailed descriptions of the technology and policy sensitivities.

Supplementary Table 2. Detailed description of scenario assumptions for technology and policy sensitivities. Each sensitivity is conducted under the assumption of a 2035 net-zero policy target.

Scenario	Abbr.	Description
**Reference**	NZ Ref	N/A
**Lower Renewables and Battery Costs**	LoRE	Capital costs for wind, solar, and batteries exhibit faster declines than the reference case, as shown in Supplementary Figure 4. Lower cost trajectories come from Bistline, et al. (2021) [14].
**Zero Emission Fossil CCS**	HiCapture	Availability of a CCS-equipped gas technology where the flue gas has CO_2 concentration similar to the atmosphere (i.e., similar to proposed Allam cycle plants). Cost and performance characteristics are assumed to be similar to a 90% capture plant.
**No New NGCC Capacity**	NoGas	No new NGCC capacity investment without CCS is allowed in any region after 2020. Construction of gas-fired peaking units is permitted.
**No New NGCC or CCS Capacity**	NoGasCCS	No new NGCC or CCS-equipped capacity (including gas, coal, and biomass) is allowed in any region after 2020. Construction of gas-fired peaking units is permitted.
**Upstream Methane with 3% Leakage**	3% Leak	A higher upstream CH_4 leakage rate of 3% is used to represent worst-in-class performance [15]. Note that these sensitivities with static emissions coefficients likely represent upper bounds on CH_4 impacts, since mitigation measures can lower leakage rates.
**CCS Tax Credits**	45Q	Section 45Q tax credits are available beginning at \$32/t-CO_2 for sequestered CO_2 in 2020 and increasing to \$50/ t-CO_2 by 2026 for no more than 12 years for units that are under construction by 2024.
**Low-Cost Long-Duration Energy**	LDES	In addition to hydrogen, compressed air, and pumped hydro in the other runs, this sensitivity includes the availability of a stylized long-duration

**Storage**		storage technology with energy capacity costs of $10/kWh and power costs of $400/kW. These values are consistent with the U.S. DOE's "Long Duration Storage Shot" and the competitiveness threshold identified in Sepulveda, et al. (2021) [16].
**Pessimistic Natural Gas Assumptions**	Pess	This sensitivity combines pessimistic assumptions about gas from earlier sensitivities—including high CH_4 leakage, high prices, high BECCS cost ($10,000/kW), no DAC, and high CO_2 storage costs (doubling reference costs)—with optimistic costs of renewables, storage, and electrolyzers.

Supplementary Note 3: Additional Electric Sector Results

Supplementary Figure 12 shows national generation and electricity prices under alternate climate policy targets and timing. In the Net-Zero scenario, carbon removal from BECCS allows gas to balance wind and solar variability, and renewables (including wind, solar, hydro, and biomass) account for roughly half of generation. In the Carbon-Free scenario, there is a large buildout of nuclear and energy storage (including both hydrogen for long-duration storage and shorter-duration battery storage) to balance larger solar and wind expansions.

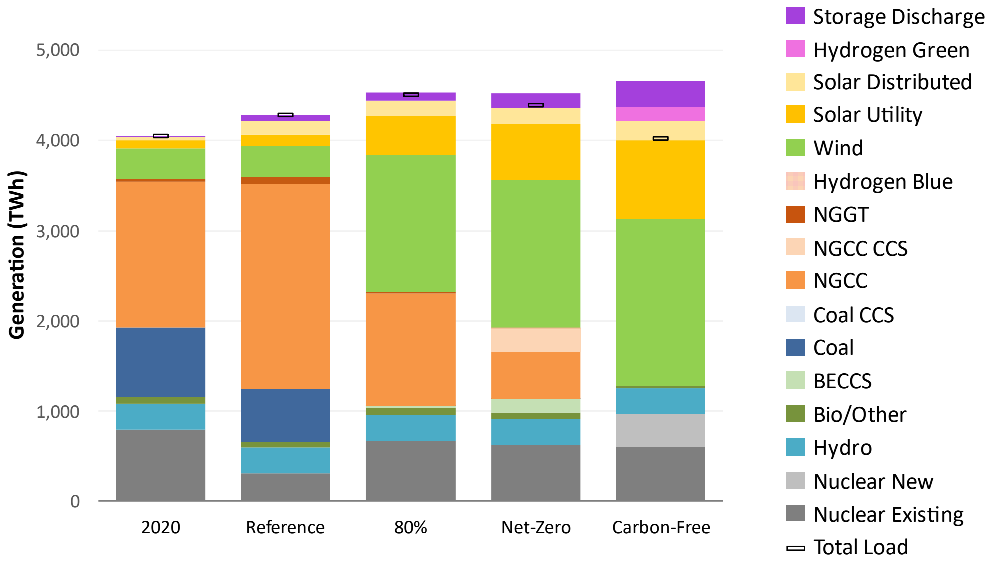

Supplementary Figure 12. National generation by technology and scenario. Generation in 2035 assuming zero-emissions targets in 2035. "80%" shows the 2035 mix with 80% reductions (with net-zero emissions in 2050). "Storage Discharge" refers to gross discharge from non-hydrogen technologies. "Total Load" refers to energy for load (i.e., consumption plus transmission and distribution losses).

Electricity price increases over the reference are modest for the Net-Zero scenario and higher for the Carbon-Free scenario. Note that these U.S. annual averages smooth out strong intra-annual and regional variability [17]. The U.S. generation-weighted average generation price is calculated by taking the hourly marginal cost of meeting demand, averaging over the year, and then over regions weighted by total generation. This metric represents all the costs covered by the electric sector model, including investment, fuel, operations, and maintenance costs for generation, bulk transmission, energy storage, and carbon removal assets, but exclude distribution and some intra-regional costs.

Supplementary Figure 13 shows regional generation shares for coal and gas over time. CO_2 policies accelerate coal retirement trends with nearly no coal generation with or without CCS beginning in 2030. Gaseous fuels have slower rates of decline owing to their lower emissions intensities and potential competitiveness of gas with CCS and hydrogen in net-zero and carbon-free systems. Generation falls faster than capacity, as dispatchable resources are used to meet growing peak demand with increasingly low capacity factors as lower CO_2 emissions are targeted (Supplementary Figure 16). There is regional variation in the rate of decline due to differences in renewable resource quality, fuel prices, existing capacity mixes, and state-level policies.

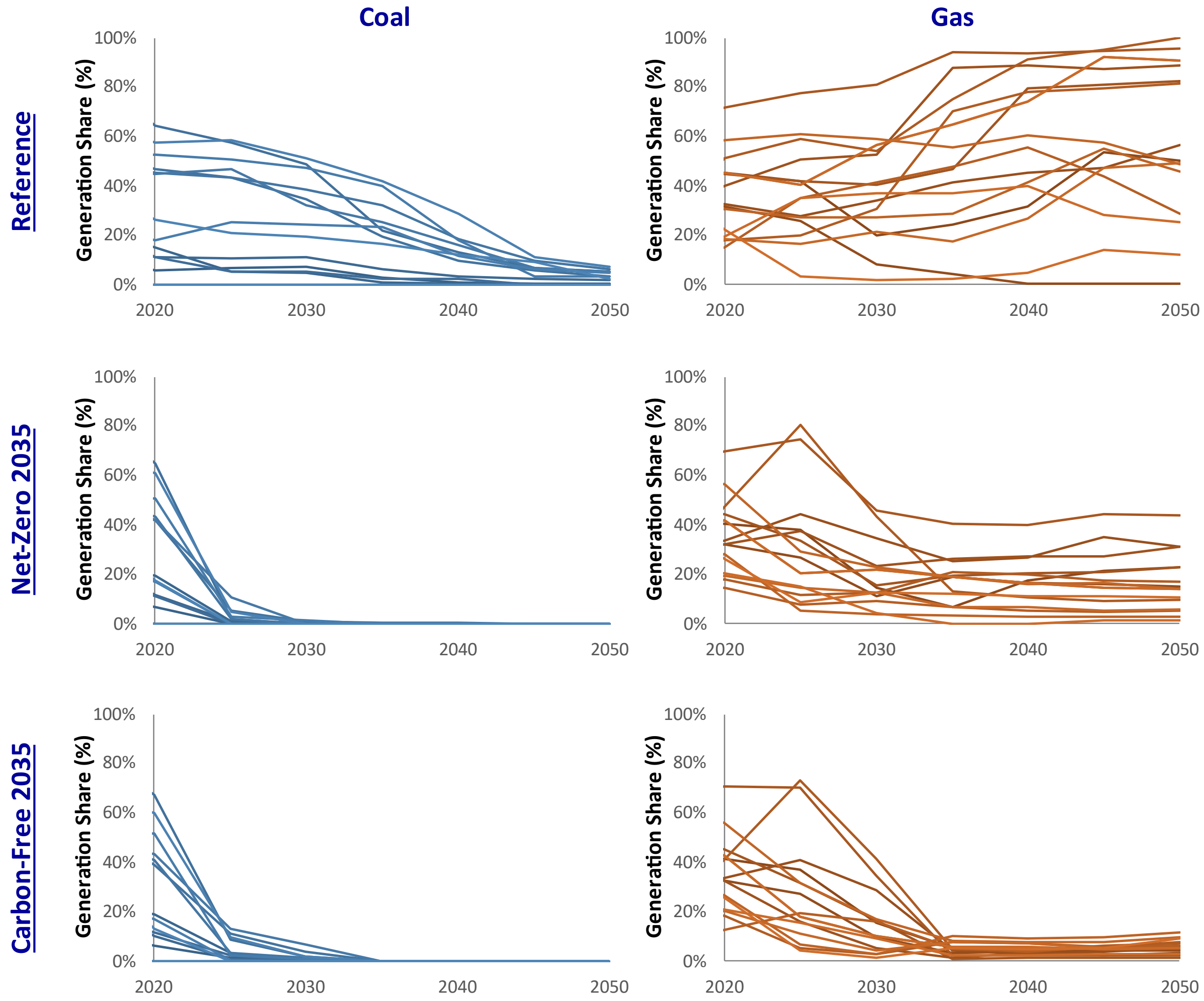

Supplementary Figure 13. Coal and gas regional generation shares over time across scenarios. Individual lines on each panel show all 16 model regions (Supplementary Figure **1**) across the three renewable cost scenarios. Coal includes all generation with and without carbon capture. Gas includes all generation with and without carbon capture and hydrogen.

Installed natural gas capacity across scenarios is shown in Supplementary Figure 14. Relative to the current fleet in 2020, many of these scenarios represent a slight increase on net or modest decreases. However, the composition of the natural gas fleet can differ across scenarios, as retirements of existing capacity are replaced by CCS-equipped units or hydrogen turbines.

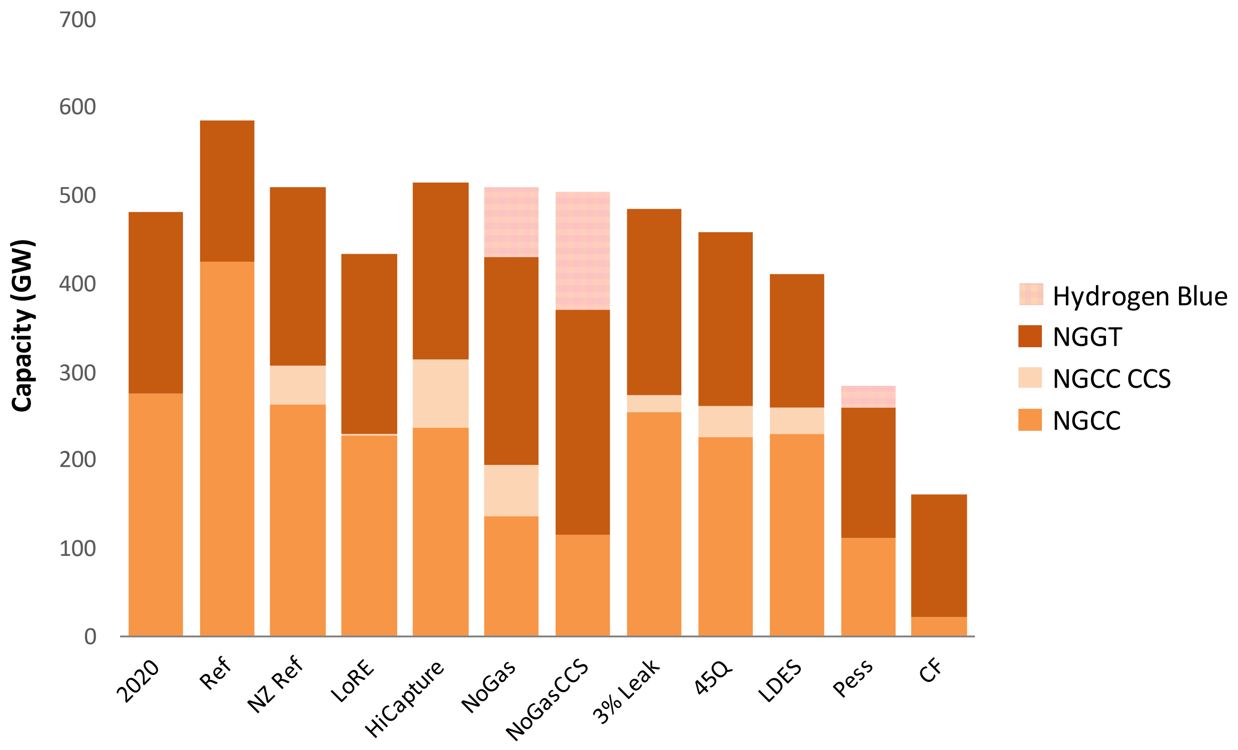

Supplementary Figure 14. Natural gas capacity by technology and scenario in 2035. Natural gas capacity includes combined cycle units without carbon capture (NGCC), NGCC with CCS (NGCS CCS), combustion turbine (NGGT), and turbines fueled by hydrogen produced from steam methane reforming with CCS (Hydrogen Blue). All scenarios except for the reference include a net-zero CO_2 by 2035 policy.

Supplementary Figure 15 illustrates positive and negative CO_2 emissions flows by technology across scenarios. The portfolio of CDR technologies deployed depends on technological cost and availability assumptions as well as the scale of demand. BECCS is deployed over DAC through 200–300 Mt-CO_2/yr; however, increasing biomass costs make DAC favorable at the margin for higher demand scenarios (e.g., reaching net-zero with high electrification). This finding is consistent with other electric sector deep decarbonization studies [9].

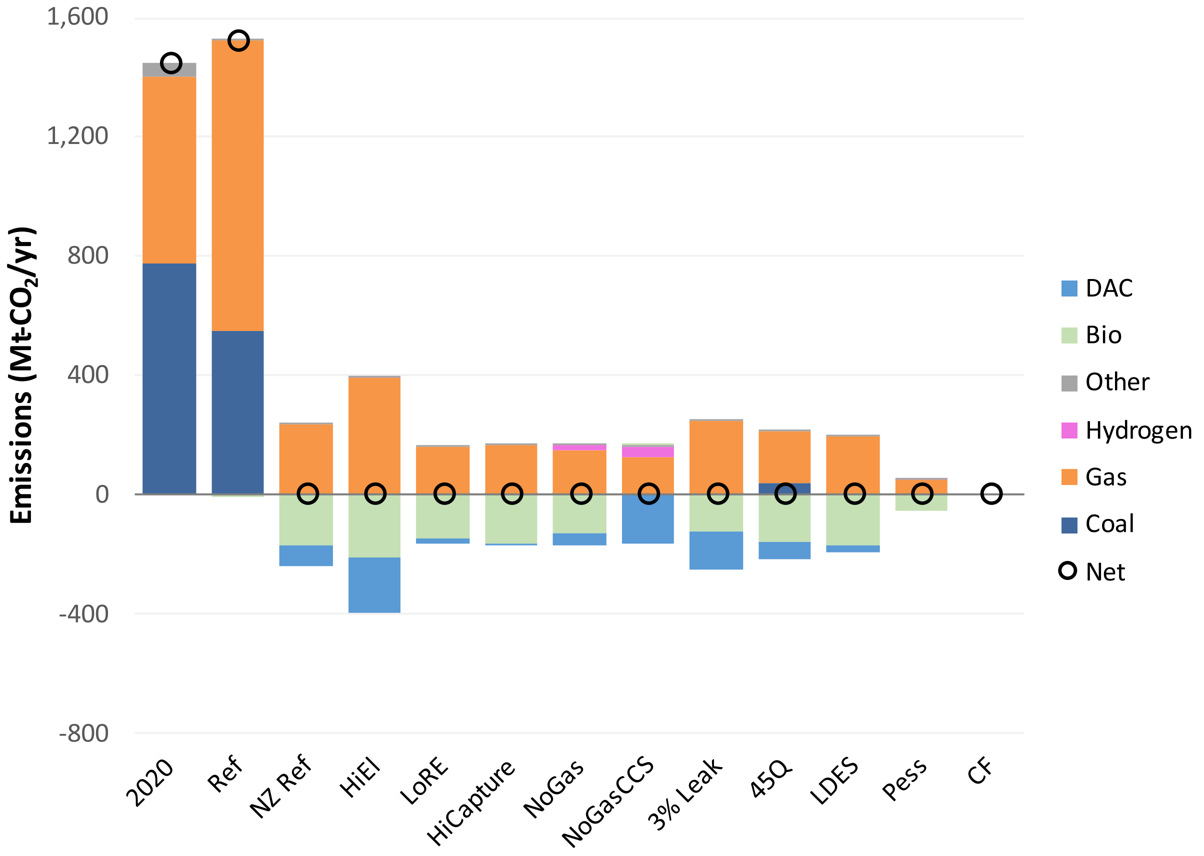

Supplementary Figure 15. CO_2 emissions by technology across different scenarios in 2035. Apart from the 2020 bar and reference scenario, all other scenarios assume a net-zero-emissions targets in 2035.

Supplementary Figure 16 illustrates capacity factors for different technologies by region and scenario. Unabated NGCC capacity factors exhibit high variability but are generally higher than gas turbines (NGGT) or blue hydrogen. NGCC with CCS plays a different role than uncaptured gas: The former is a higher capital cost and higher capacity factor option, which is preferred to new nuclear but only deployed when it can operate with capacity factors above roughly 60% to balance wind and solar, after which uncaptured gas of some form is preferred [18]. Supplementary Figure 16 shows variation in regional capacity factors over time due to different existing capacity mixes, renewable resources, policies, and gas prices. For a Net-Zero by 2035 policy, capacity factors generally fall by 50% between 2020 and 2035.

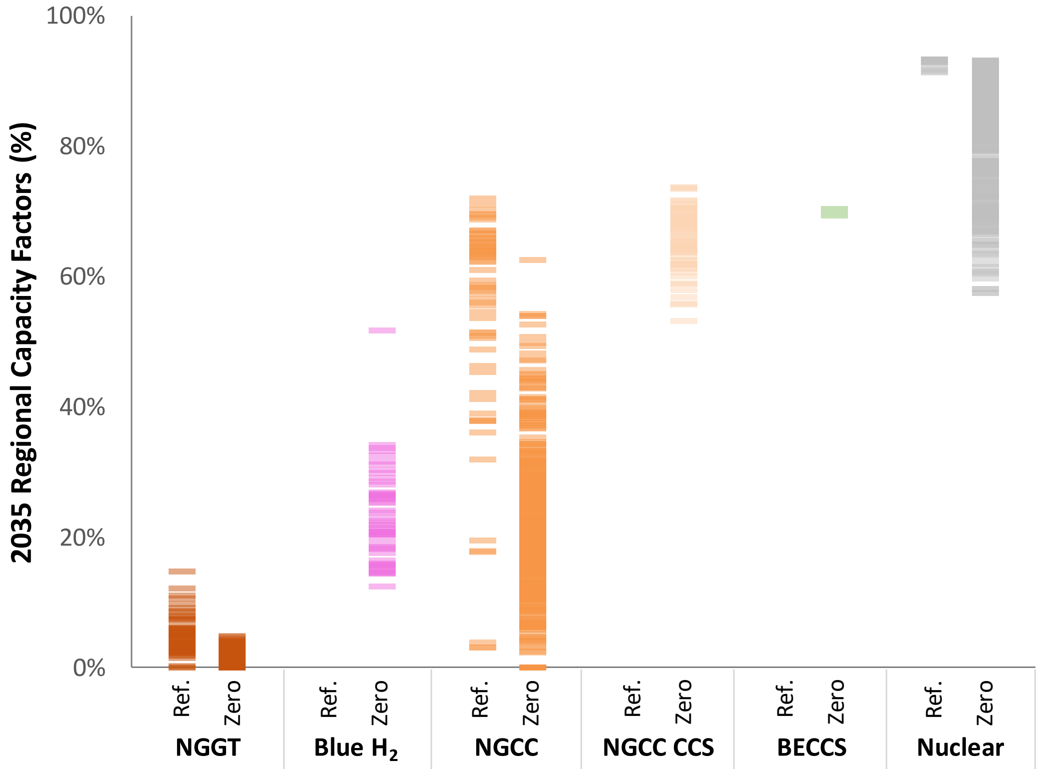

Supplementary Figure 16. Regional capacity factors by technology in 2035. Columns show different policy scenarios. Dashes show capacity factors across all regions and scenarios.

Supplementary Figure 17 shows how gas helps to balance the system across different timescales due to daily, weekly, and seasonal profiles from electrification and variable renewable output.

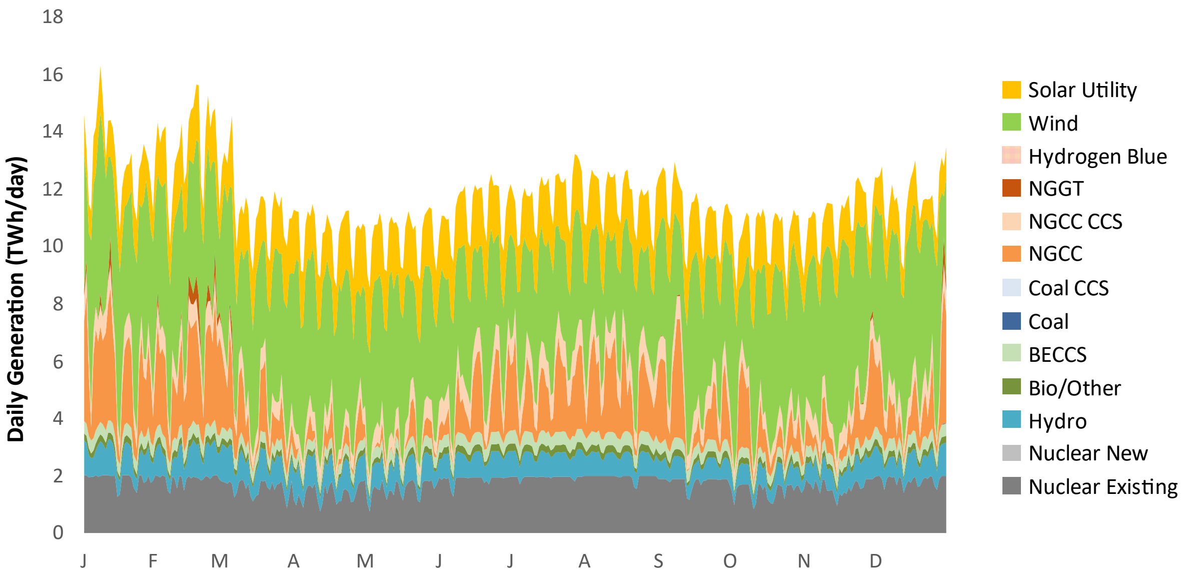

Supplementary Figure 17. Daily electric sector generation by technology. Values are shown for the Net-Zero by 2035 scenario in 2035.

Targeting zero emissions by 2050 (instead of 2035) in the electric sector entails higher deployment of solar and battery storage and lower CCS-equipped gas, wind, and new nuclear.

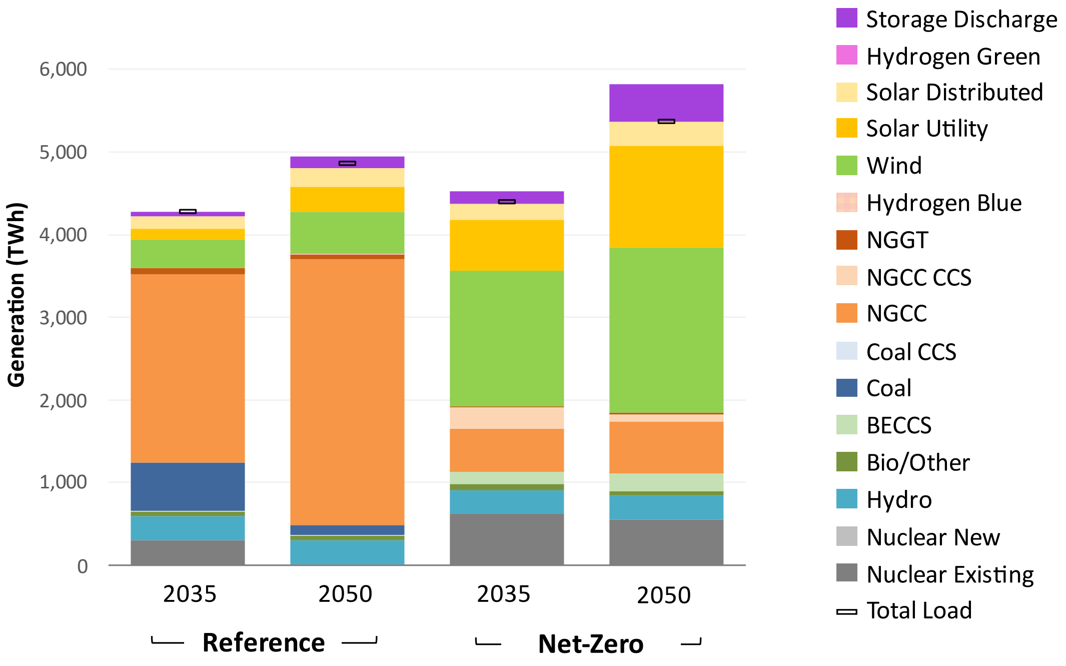

Supplementary Figure 18. National electric sector generation by technology and scenario in 2035 and 2050. "Net-Zero" scenarios vary whether the target is reached in 2035 or 2050.

Supplementary Figure 19 shows normalized revenues for existing NGCC capacity, which are proxies for the market value of capacity. Energy revenues refer to bulk electricity sales, and capacity revenues relate to the ability to provide firm capacity contributions during periods of system stress. In capacity planning optimization models, energy revenues typically come from the dual variable on the hourly market-clearing constraint for electricity, and capacity revenues are from the planning reserve margin constraint. Under reference policy conditions, energy revenues are the dominant value stream for NGCC plants in many regions. However, with the Net-Zero by 2035 policy, revenues shift from energy to capacity, but overall returns are much lower with more stringent policy. There is considerable regional heterogeneity in the magnitude of this asset impairment, which varies based on regional natural gas prices, the existing asset mix, renewable resource characteristics, and extent of existing plant retirements. Note that the model endogenously retires existing capacity when the net present value of going-forward costs exceeds that of projected revenues in a given scenario. These results suggest the importance for electricity market structures to effectively value capacity in deeply decarbonized systems.

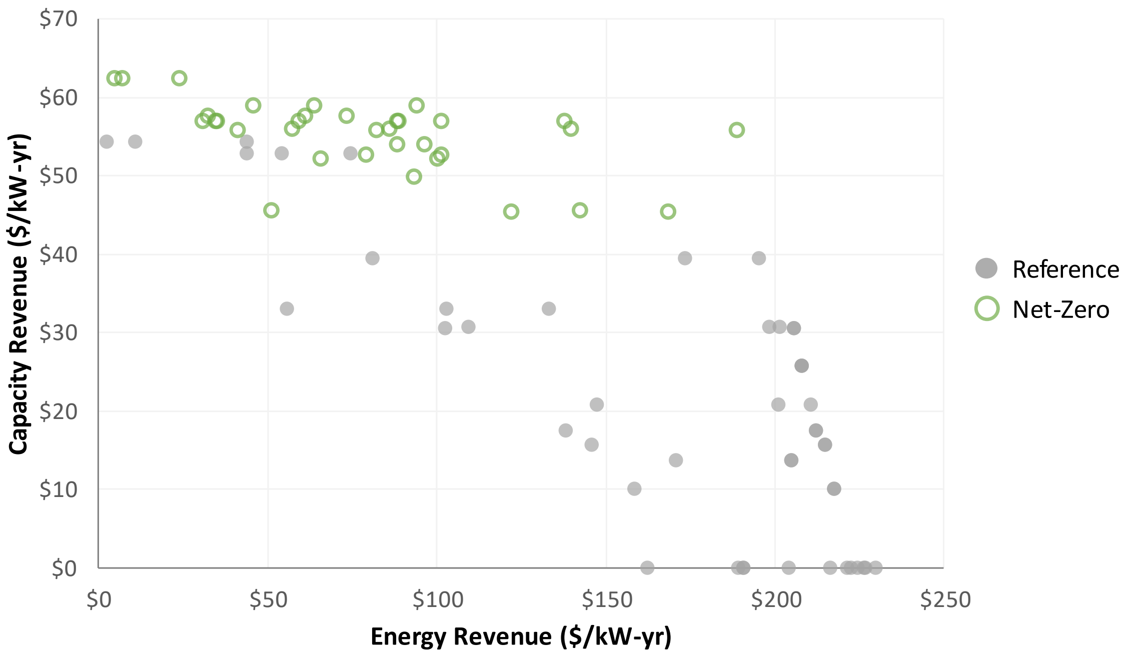

Supplementary Figure 19. Regional energy and capacity revenues ($ per kW-year) for existing natural gas capacity in 2035. Points show values across the 16 model regions (Supplementary Figure 1) for each of the three existing NGCC capacity classes. Values are shown for the Reference and Net-Zero by 2035 scenarios.

To illustrate the economics of natural gas additions with deeper CO_2 reductions, Supplementary Figure 20 shows revenues and costs over time for a hypothetical NGCC plant built in 2025 under the Net-Zero by 2035 scenario. Upfront investment costs are shown for the year the plant comes online (2025). Net operating revenue is relatively flat over time due to the firm back-up role for variable renewables, as gas typically sets the price in energy markets. A large fraction of revenues (83% for this scenario and region) occur in the first 10 years of operation.

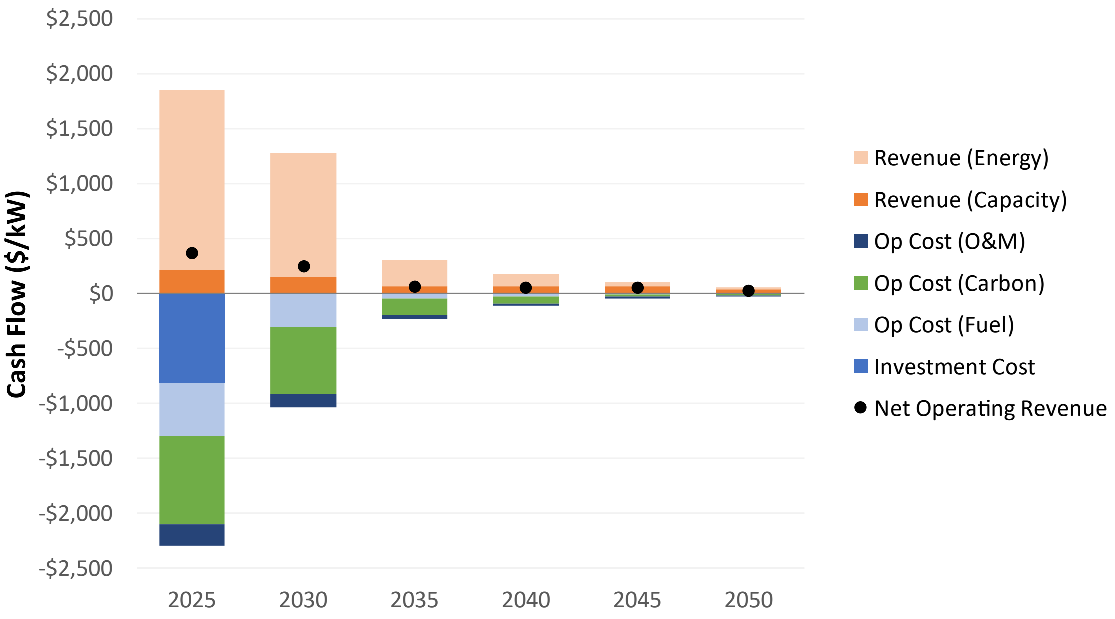

Supplementary Figure 20. Revenues and costs for an illustrative NGCC plant over time in the Net-Zero by 2035 scenario. All cash flows are shown in discounted terms for a 2025 vintage NGCC plant in the Mid-Atlantic model region.

Note that comparing the capital cost of new renewables with the operating cost of gas does not provide a complete picture of economic competitiveness or stranded asset risk. Studies comparing levelized costs of wind and solar with operational costs of dispatchable fossil units are not comparing like-for-like quantities. Supplementary Figure 21 illustrates how cost metrics only tell part of the story, since revenues are quite different across asset classes. Although levelized costs of solar are indeed lower than the fuel and operating costs for NGCC capacity, normalized revenues are much higher for NGCC capacity, even with lower capacity factors.

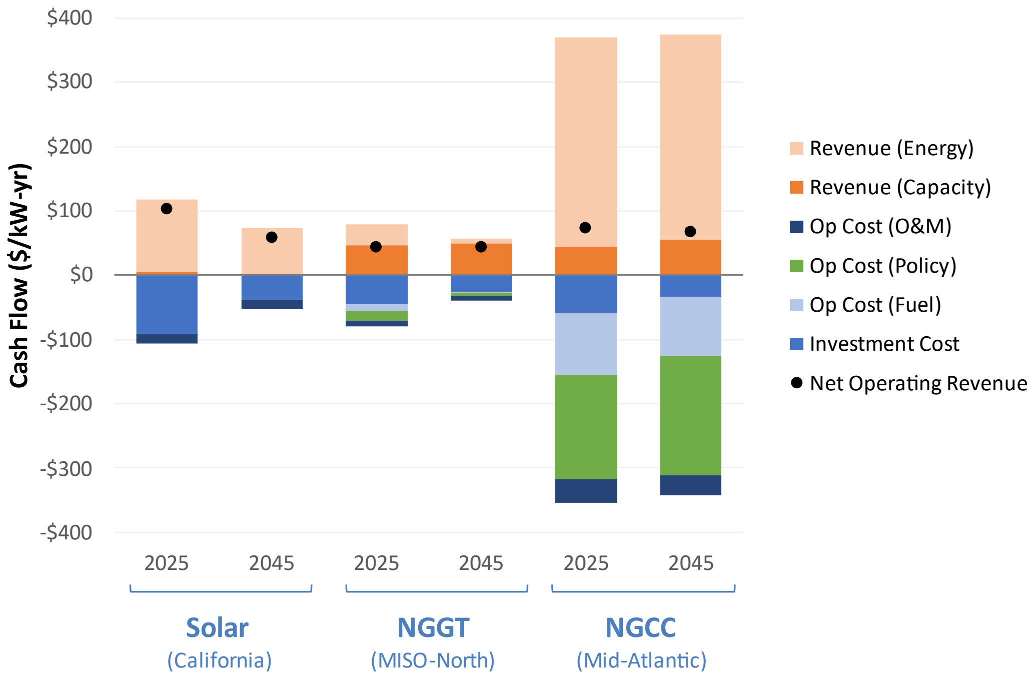

Supplementary Figure 21. Revenues and costs across technology types in the Net-Zero by 2035 scenario. All cash flows are shown in discounted terms for 2025 and 2045 vintages. Investment costs show the upfront cost amortized over the lifetime of the capacity.

To test the robustness of results to alternate technological assumptions, Supplementary Figure 22 shows the generation mix and direct air capture capacity across several sensitivities:

- **Net-Zero:** Net-zero electric sector CO_2 emissions by 2035 with reference technology assumptions. This is the same Net-Zero scenario used earlier in the paper.
- **Low-Cost Long-Duration Energy Storage (LDES):** Availability of a stylized long-duration storage technology with energy capacity costs of $10/kWh, which is consistent with the U.S. DOE's "Long Duration Storage Shot." This technology is in addition to other energy storage technologies represented in the model, including batteries, compressed air energy storage, existing pumped hydro, and hydrogen via electrolysis.
- **Low Hydrogen:** The net-zero policy scenario is paired with low hydrogen costs, where lower electrolysis system costs are assumed (Supplementary Figure 7).
- **DAC Only:** The net-zero policy is met without the availability of BECCS, and any carbon removal must be provided by DAC.
- **No New NGCC or CCS Capacity (NoGasCCS):** No new NGCC or CCS-equipped capacity (including gas, coal, and biomass) is allowed in any region after 2020.
- **High-Cost BECCS:** This net-zero scenario examines a case where DAC is not allowed and BECCS capital costs are high across the time horizon (assumed to be $10,000/kW instead of the values in Supplementary Figure 4).
- **Carbon-Free (CF):** This scenario assumes that zero emissions are reached without the use of fossil fuels or carbon removal.

Supplementary Figure 22 shows how the natural gas generation share varies across these technological scenarios. BECCS is deployed in many net-zero scenarios, but when it is unavailable (“DAC Only” and “NoGasCCS”), then DAC deployment is higher alongside greater deployment of CCS on gas generation. When DAC is constrained and BECCS costs are high (“High-Cost BECCS”), more natural gas with CCS appears in the generation mix with lower unabated NGCC generation, which is complemented by blue hydrogen (i.e., hydrogen produced from steam methane reforming with CCS). The only scenario with green hydrogen (i.e., hydrogen produced from electrolysis with zero-emissions electricity) and without natural gas is the Carbon-Free scenario, where fossil fuels and carbon removal are explicitly prohibited.

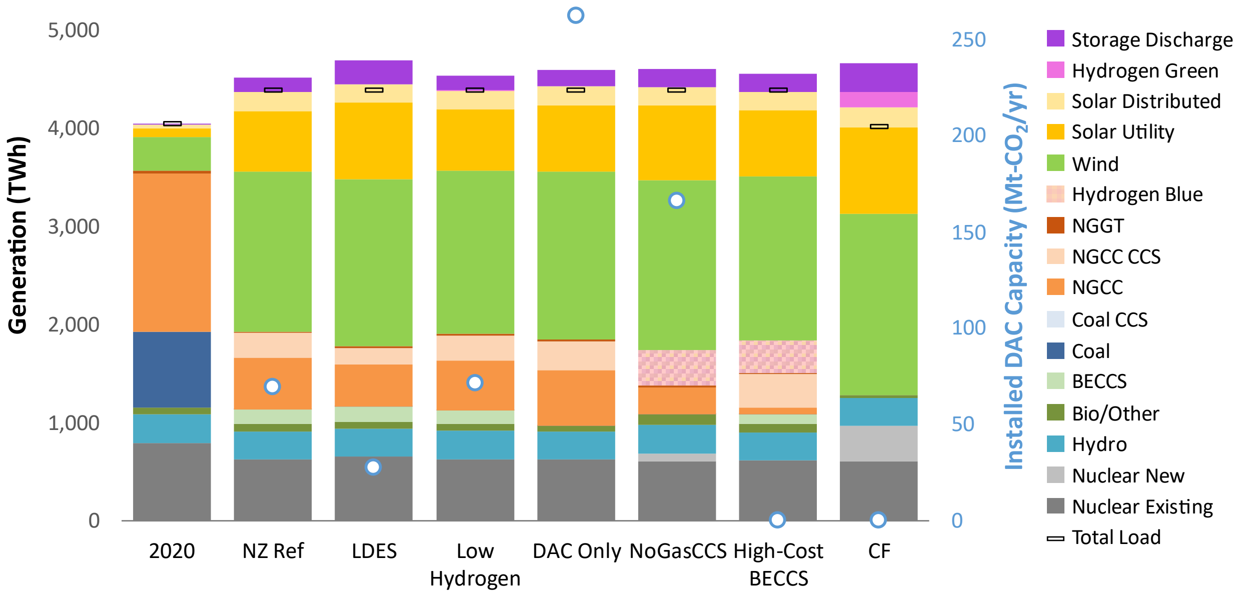

Supplementary Figure 22. National generation by technology and scenario in 2035. Generation assuming zero-emissions targets in 2035.

Biomass supply and use in net-zero scenarios with reference and high electrification are shown in Supplementary Figure 23. Biomass consumption from BECCS deployment is 2.46 and 3.06 quads under the net-zero scenarios with reference and high electrification, respectively. Biomass consumption in the U.S. in 2020 totaled 4.5 quads with about 0.4 quads in the electric sector, so BECCS at this scale represents an increase of 45–59% in economy-wide biomass consumption. For context, the Princeton “Net-Zero America” study indicates that, for economy-wide net-zero emissions scenarios, biomass consumption is between 11.7–21.7 quads [19].

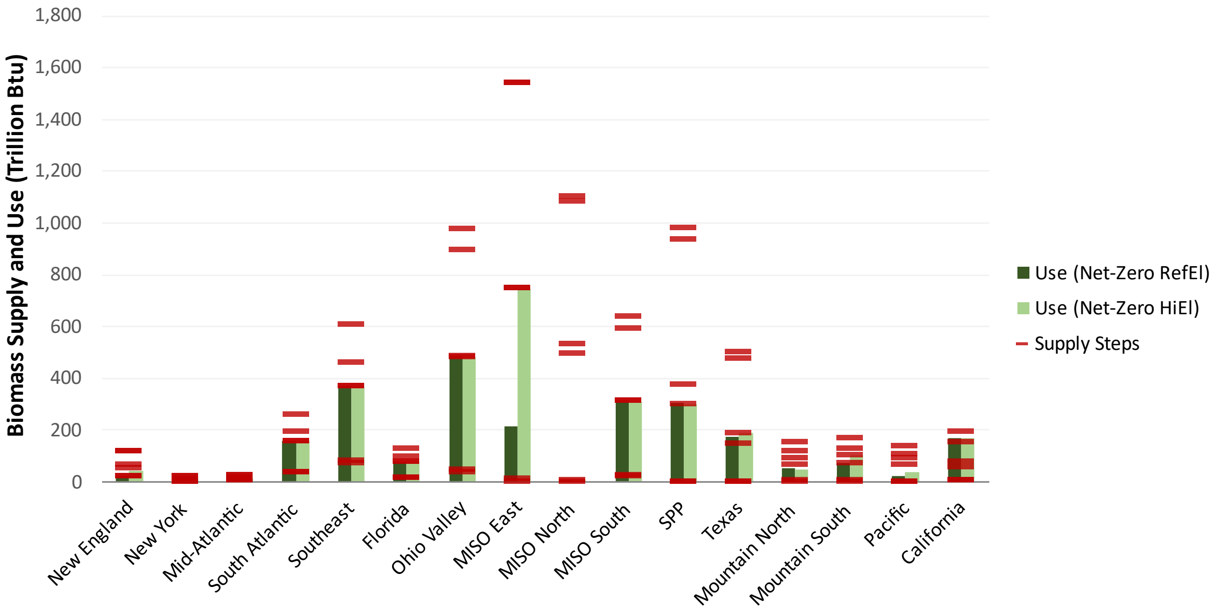

Supplementary Figure 23. Biomass supply and use by region in 2035. Values shown for the Net-Zero in 2035 scenarios with reference and high electrification (RefEl and HiEl, respectively). Cumulative biomass supply by step in the piecewise linear supply curve are shown as red dashes.

A sensitivity examines how the availability of a higher capture rate gas with CCS technology (similar to proposed Allam cycle plants) could impact investments and costs. This stylized sensitivity assumes similar cost and performance characteristics as the 90% capture plant with greater than 99% CO_2 capture so that flue gas has CO_2 concentrations less than the atmosphere. In the net-zero by 2035 scenario, the availability of higher capture rate CCS increases capacity deployment from 44 GW to 77 GW, which includes expansion in more regions of the country. The availability of high capture rate generation has a larger impact under a "Carbon-Free" policy formulation, as illustrated in Supplementary Figure 18. Under this policy formulation, gas becomes a larger part of the mix and displaces generation from new nuclear, battery storage, long-duration energy storage, and renewables. Higher capture rate CCS also lowers electricity prices from $68/MWh to $64/MWh under the net-zero policy and from $76/MWh to $69/MWh under the carbon-free policy.

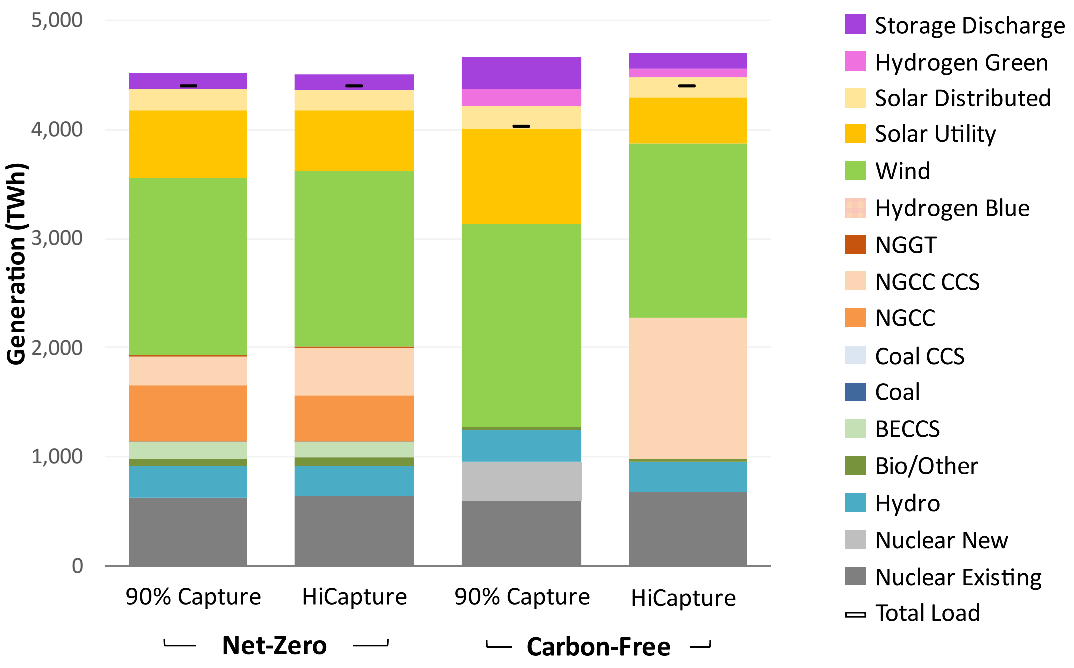

Supplementary Figure 24. National electric sector generation by technology and scenario in 2035 under different policy and CCS capture rate assumptions. All scenarios assume "Net-Zero" or "Carbon-Free" policies in 2035.

We also ran sensitivities where we took the objective function value from core runs; constrained costs in a new run to be within 2%, 4%, and 6% of the objective function value; and minimized and maximized the amount of NGCC capacity without CCS. Supplementary Figure 25 shows how the substitutability of gas capacity depends on opportunity costs. New NGCC investments can be avoided with a less than 2% increase in total costs, and under these conditions, generation and capacity increase from CCS-equipped gas, existing gas, and battery storage. Overbuilding NGCC capacity with CO_2 target has symmetrical cost impacts. This sensitivity indicates that a range of new gas builds could be consistent with climate goals and that region- and country-specific generation portfolios can vary significantly while keeping overall system cost near the minimum objective function value.

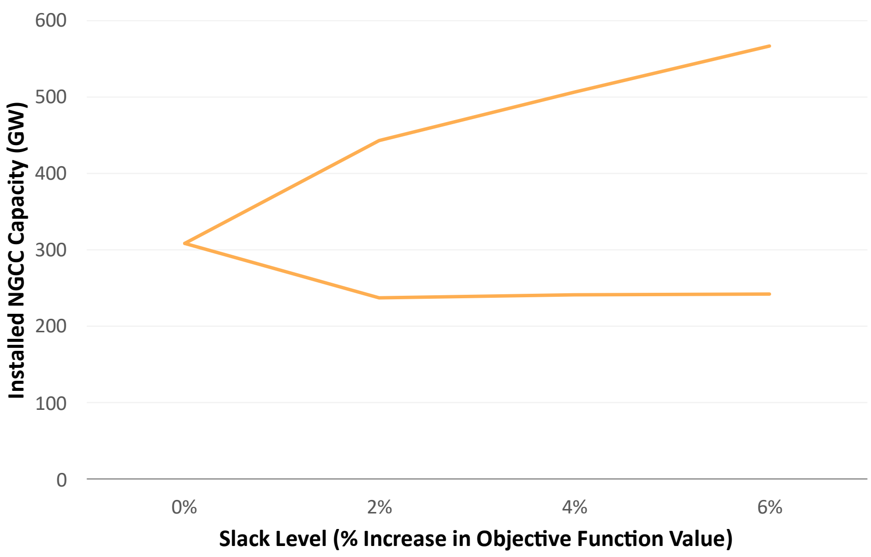

Supplementary Figure 25. Comparison of installed NGCC capacity across different slack levels. All scenarios assume a net-zero by 2035 policy.

Supplementary Figure 26 shows how higher and lower natural gas price trajectories (Supplementary Figure 11) impact the generation mix. In the reference scenario, there are countervailing CO_2 impacts of low gas prices, which simultaneously push out coal but also existing nuclear. With net-zero policies, gas prices primarily impact the degree of CCS deployment.

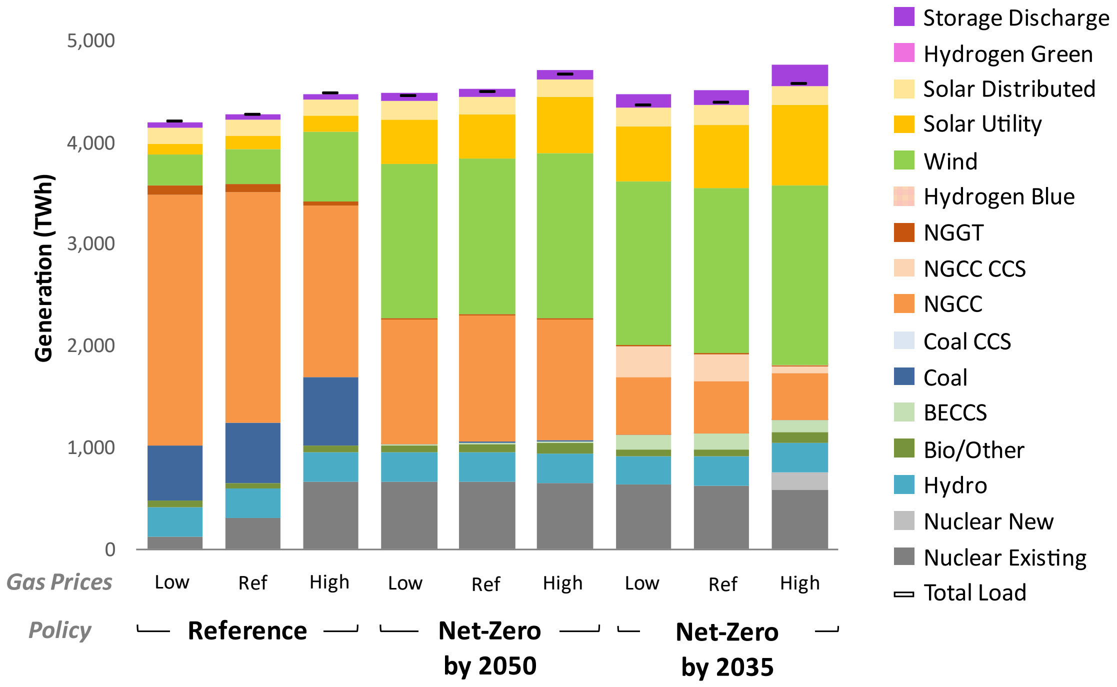

Supplementary Figure 26. National generation by technology and scenario in 2035. Mixes are compared across policy and natural gas price sensitivities.

Supplementary Note 4: End-Use Results

Two alternate electrification scenarios are run to explore how end-use demand affects the role of natural gas under different electric sector CO_2 emissions targets:

- **Reference electrification (RefEl):** Earlier results focused on scenarios that assumed reference levels of end-use electrification. In scenarios with electric sector CO_2 policies, federal CO_2 pricing of $50/t-$CO_2$ is assumed in all non-electric sectors and regions beginning in 2025, escalating at 7% per year, which is intended as a proxy for a suite of CO_2 policies for end-use sectors. Reference end-use technological and behavior assumptions are used, as documented at: https://us-regen-docs.epri.com
- **High electrification (HiEl):** This scenario increases the stringency of the CO_2 price to $100/t-$CO_2$ in 2025, escalating at 10% per year. This scenario also assumes additional technology and policy drivers that accelerate electrification by lowering the cost of end-use technologies, reducing customers' reticence to shift technologies, and accelerating the turnover of equipment. Accelerated cost reductions in this scenario could be interpreted either as faster-than-expected cost declines or as policy-driven incentives.

Each electrification scenario is run assuming an electric sector net-zero CO_2 target. Load management is incorporated through deferrable electric vehicle charging. Hourly load shapes for different end-use applications and structural classes are discussed in Bistline, et al. (2021).

Supplementary Figure 27 illustrates electricity demand by end-use application over time across different combinations of electrification assumptions and electric sector policies. Even in the reference case without additional policies, electrification of transport and industry are extensive, as the lower capital and operating costs of these options lead to greater adoption over time. Transportation leads electricity demand growth in the reference electrification scenarios with extensive light-duty vehicle electrification and non-passenger transport electrification that increases when a CO_2 tax is added. Although building electricity demand increases for space and water heating, efficiency improvements offset electrification in many instances, especially in scenarios with higher electricity prices. For instance, higher electricity prices under the Carbon-Free scenario lead to lower electricity demand relative to the Net-Zero case. Note that electricity demand drops in 2035 due to the electric sector policy requiring a 80% CO_2 reduction (Supplementary Figure 3). Industrial electrification increases in policy scenarios and dominates load growth in the high electrification scenario.

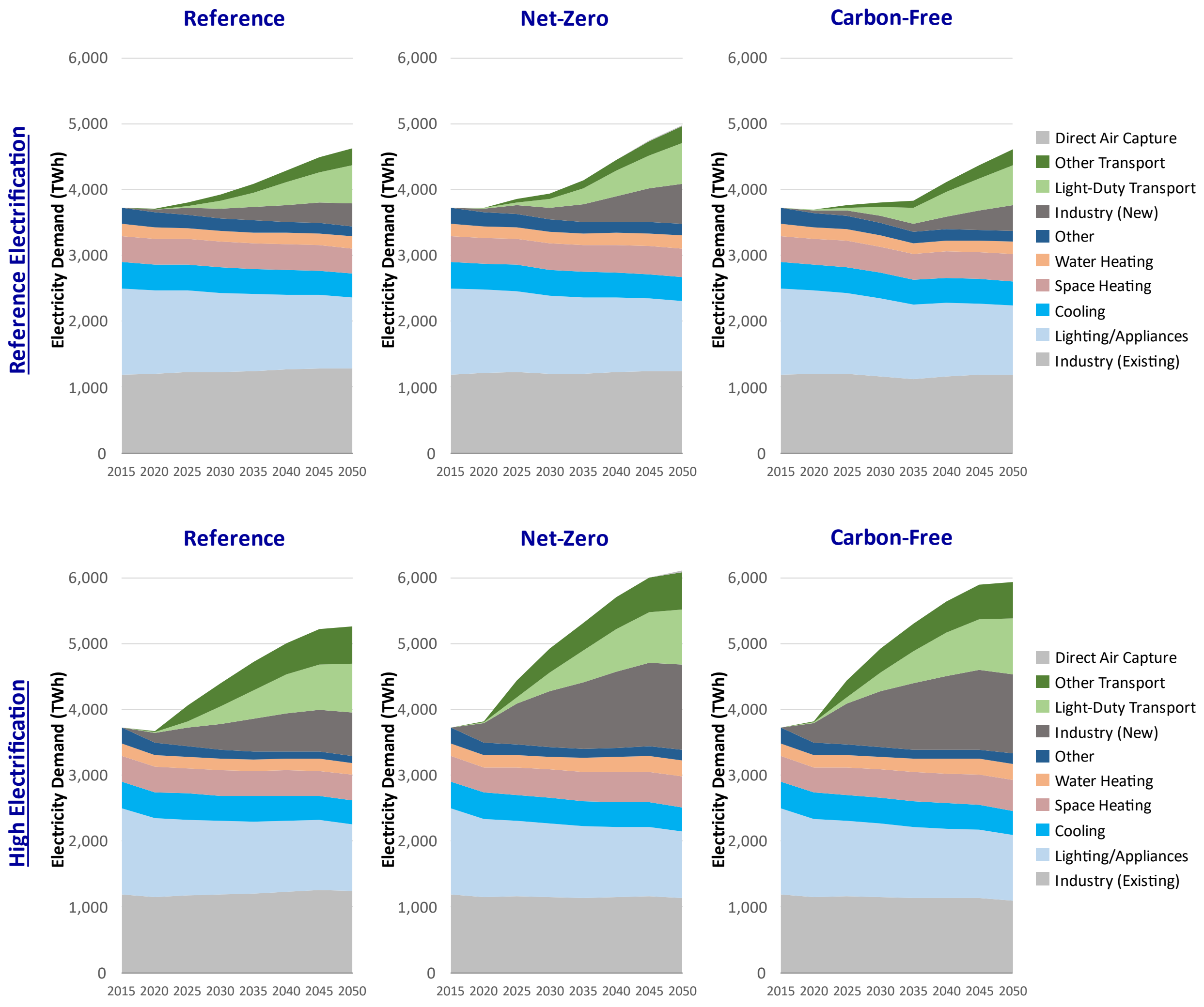

Supplementary Figure 27. Electricity demand by end-use application over time across scenarios. Electricity demand comes from the US-REGEN end-use model described in Notes S1 and S2. Rows represent the reference and high electrification scenarios with columns representing the electric sector policy scenarios.

Electrification and efficiency change both seasonal and diurnal load shapes, though impacts vary by policy and region (Supplementary Figure 28). Electrification of space heating with higher heat pump deployment leads to higher wintertime peaks, which are exacerbated by colder climates. Hourly load shapes changes from space heating are dependent on assumptions about technological performance, adoption of supplemental heating fuels, and equipment sizing. Supplementary Figure 28 illustrates that space heating electrification increases peak demand in New England with its cold climate but a relatively modest impact in California, which has a range of climate zones including many mild ones. Electric vehicle charging is a large portion of load in all regions and scenarios. Lower cold-weather charging efficiency contributes to higher charging load in the coldest hours, though this effect is partially mitigated by coordinated charging.

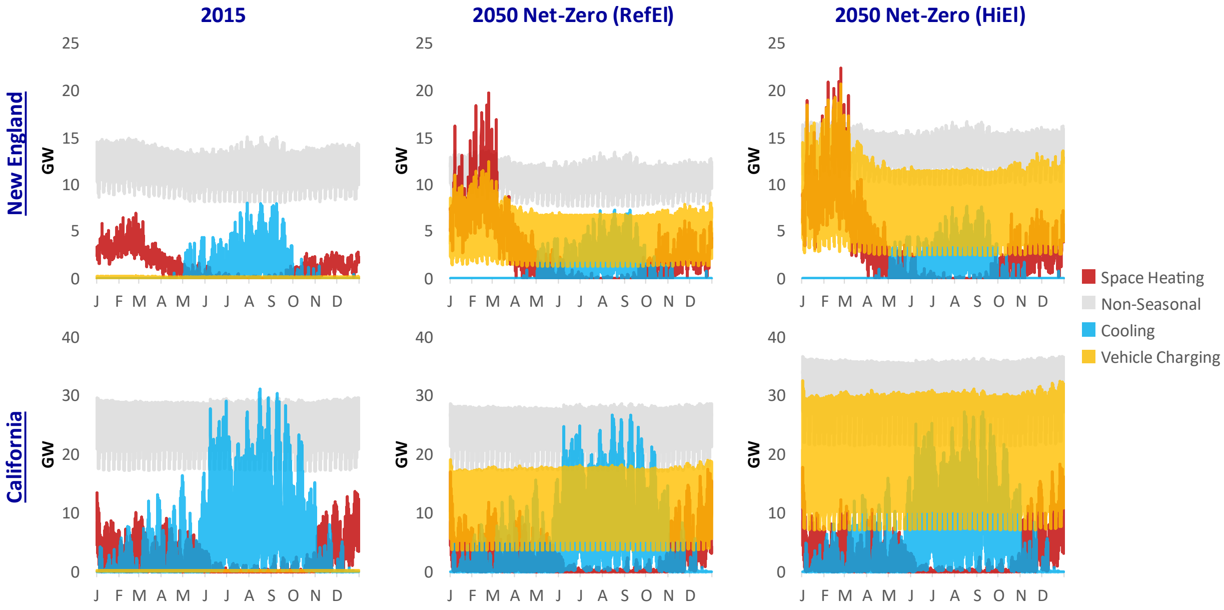

Supplementary Figure 28. Load profiles by end use across scenarios (columns) and regions (rows). Non-seasonal loads include all non-space-conditioning loads in buildings as well as industrial loads.

Electrification rates as a share of final energy for these scenarios in 2035 and 2050 generally align with recent economy-wide deep decarbonization and net-zero scenarios (Supplementary Figure 29). Electricity's share of final energy increases in the reference electrification scenario from 21% today to 28% in 2035 and then to 40% by 2050. The higher electrification scenarios reach 44% in 2035 and 65% in 2050. Although electricity demand increases with higher end-use electrification, load growth exhibits a broader range. There are three interacting policy-induced effects that lead to differences in electricity demand across models, scenarios, and countries:

- Policies such as CO_2 taxes alter end-use energy prices, which influence fuel demand contingent on policy design and emissions intensities.
- Electricity prices drop relative to fossil fuels due to the greater range of mitigation technologies in the power sector and their relatively low costs, which leads to electrification—the substitution of electricity for fossil fuel use. This leads to higher electricity demand.
- Challenging-to-electrify end uses such as heavy industry and air travel could lead to indirect electrification through electrolytic hydrogen and synthetic hydrocarbon fuels that are derived from electricity. Such indirect electrification can increase load growth, especially since such fuel conversion pathways typically entail low roundtrip efficiencies. However, such alternative fuels face competition from direct electrification, alternative low-emitting fuel pathways (e.g., hydrogen from steam methane reforming with carbon capture, biofuels), and using fossil fuels with carbon removal.

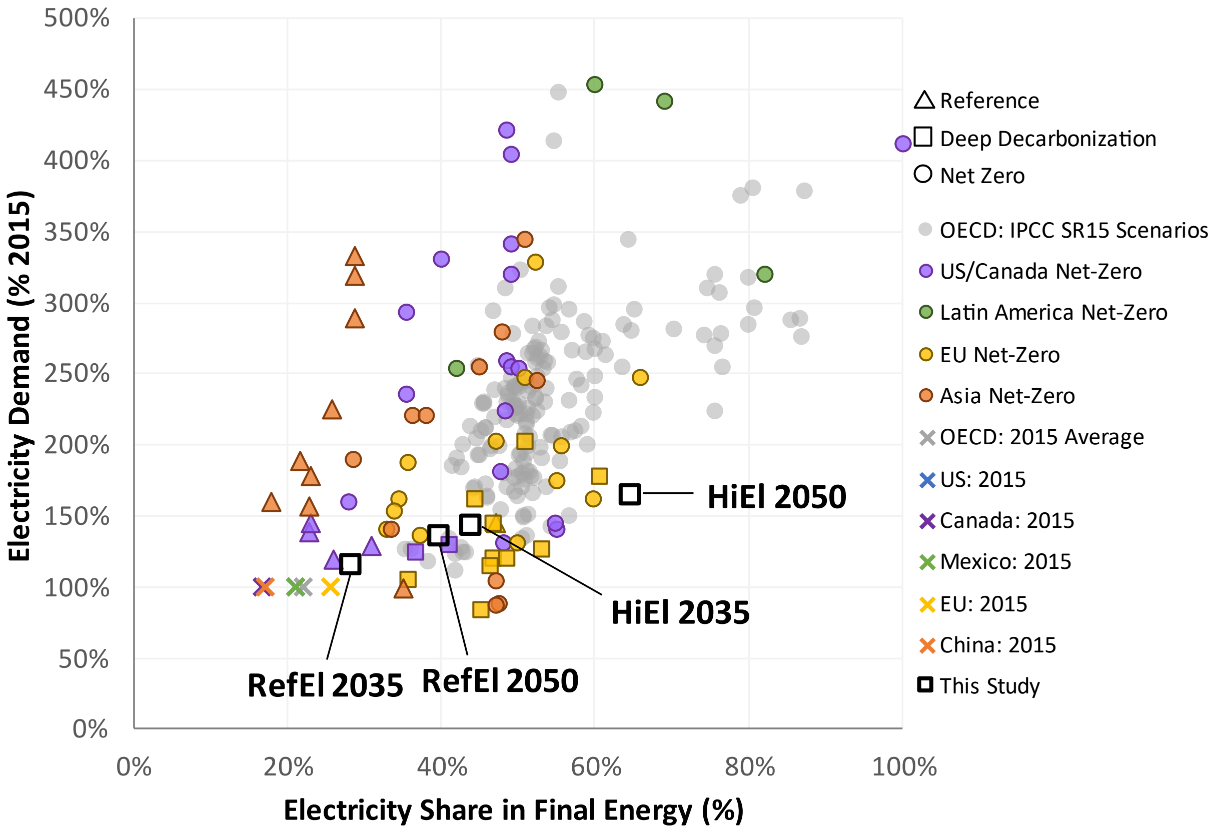

Supplementary Figure 29. Differences in electrification and electricity demand growth across scenarios. Points represent individual scenarios with shapes corresponding to emissions levels (triangle, reference scenario in 2050; square, deep decarbonization in 2050; circle, net zero in 2050), and colors correspond to different analysis regions. Detailed descriptions are provided in Bistline (2021).

US-REGEN scenarios are on the lower end of deep decarbonization scenarios for several reasons. First, many scenarios in the analysis focus on reaching zero emissions in the power sector by 2035, and electrification and load growth are generally higher by 2050. Second, end-use scenarios in this analysis have significant emissions reductions relative to current levels or the reference (Supplementary Figure 30), but the analysis does not look at economy-wide net-zero targets, which entail higher electrification. Finally, US-REGEN includes greater contributions from exogenous and endogenous energy efficiency, which place its demand growth closer to EU-based models rather than US-based ones that are dominated by models that have more limited representations of energy efficiency [20].

This electrification leads to substantial economy-wide CO_2 emissions reductions (Supplementary Figure 30). Under reference scenario conditions, 2050 CO_2 decreases 44% from 2005 levels with reference electrification and 55% with higher electrification. The Net-Zero and Carbon-free cases lower 2050 CO_2 emissions by 70% and 71%, respectively, under the reference electrification scenario and 88% with higher electrification.

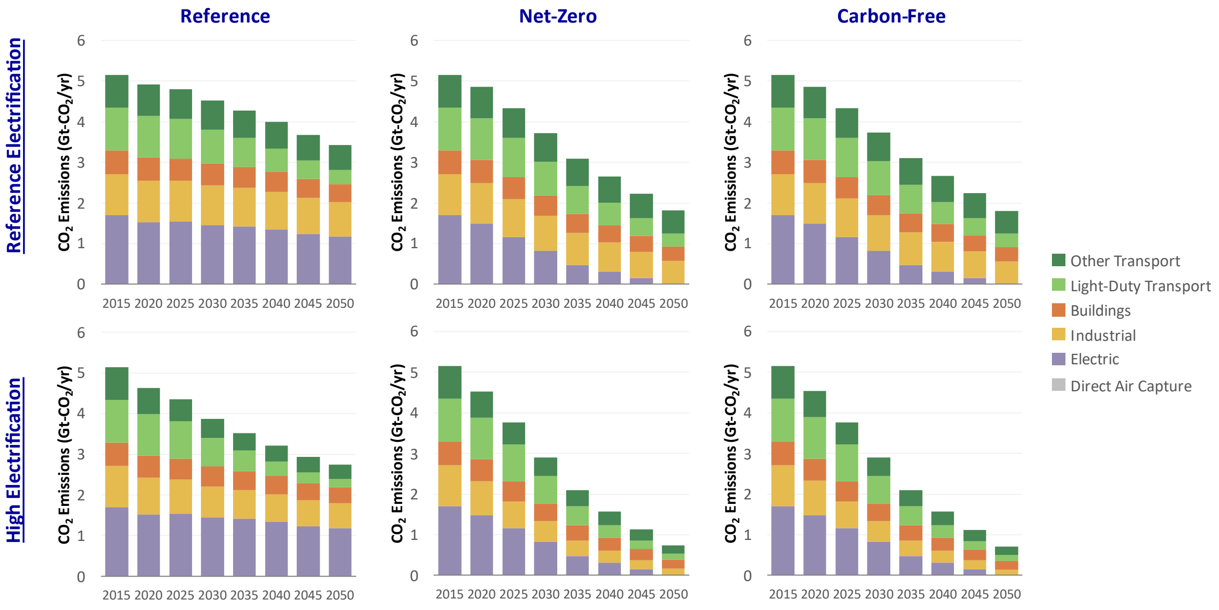

Supplementary Figure 30. Sectoral CO_2 emissions across electrification scenarios (rows) and electric sector policy scenarios (columns) over time. Land sink and non-CO_2 GHGs are not shown.

CO_2 policy lowers natural gas demand in electric and non-electric sectors, as shown in Supplementary Figure 31. Variation across gas price scenarios is lower than for policy. With more restrictive net-zero definitions ("Carbon-Free"), electricity price increases disincentivize electrification, and hence increasing non-electric gas demand. This de-electrification rebound effect implies that partial equilibrium models of the power sector may understate emissions by omitting such leakage channels [21].

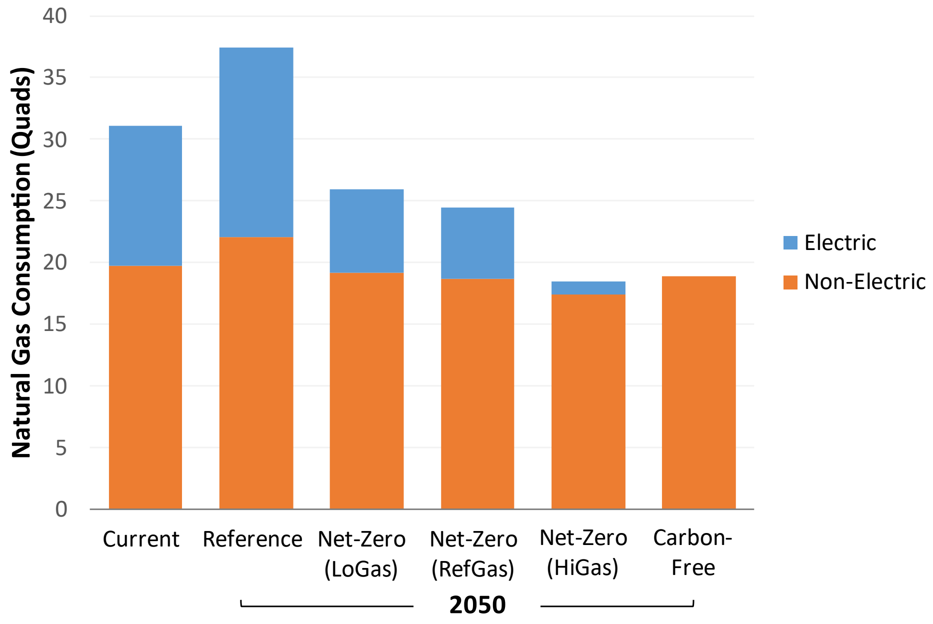

Supplementary Figure 31. Economy-wide natural gas consumption in 2050. Gas use is shown for electric and non-electric end uses.

Supplementary References

[1] EIA, "Annual Energy Outlook with Projections to 2050," U.S. Energy Information Administration, Washington, DC, 2020.

[2] EPRI, "US-REGEN Model Documentation," Electric Power Research Institute, Palo Alto, CA, 2020.

[3] J. Bistline, C. Roney, D. McCollum and G. Blanford, "Deep Decarbonization Impacts on Electric Load Shapes and Peak Demand," *Environmental Research Letters,* vol. 16, no. 9, 2021.

[4] J. Bistline, W. Cole, G. Damato, J. DeCarolis, W. Frazier, V. Linga, C. Marcy, C. Namovicz, K. Podkaminer, R. Sims, M. Sukunta and D. Young, "Energy Storage in Long-Term System Models: A Review of Considerations, Best Practices, and Research Needs," *Progress in Energy,* 2020.

[5] G. Blanford, J. Merrick, J. Bistline and D. Young, "Simulating Annual Variation in Load, Wind, and Solar by Representative Hour Selection," *The Energy Journal*, vol. 39, no. 3, p. 183–207, 2018.

[6] J. Merrick, "Analysis of Foresight in Long-Term Energy System Models," EPRI, Palo Alto, CA, 2021.

[7] EPRI, "Program on Technology Innovation: Integrated Generation Technology," EPRI, Palo Alto, CA, 2018.

[8] E. Minear, "Battery Energy Storage Installation Cost Estimation Tool Version 1," EPRI, Palo Alto, CA, 2020.

[9] J. Bistline and G. Blanford, "Impact of Carbon Dioxide Removal Technologies on Deep Decarbonization of the Electric Power Sector," *Nature Communications,* vol. 12, p. 3732, 2021.

[10] D. Keith, G. Holmes, D. St. Angelo and K. Heidel, "A Process for Capturing CO2 from the Atmosphere," *Joule,* vol. 2, p. 1573–1594, 2018.

[11] National Academies of Sciences, Engineering, and Medicine, "Negative Emissions Technologies and Reliable Sequestration: A Research Agenda," The National Academies Press, Washington, DC, 2019.

[12] M. Fasihi, O. Efimova and C. Breyer, "Techno-Economic Assessment of CO2 Direct Air Capture Plants," *Journal of Cleaner Production,* vol. 224, pp. 957-980, 2019.

[13] N. Mac Dowell, P. Fennell, N. Shah and G. Maitland, "The Role of CO2 Capture and Utilization in Mitigating Climate Change," *Nature Climate Change*, vol. 7, no. 4, pp. 243-249, 2017.

[14] J. Bistline, R. Bedilion, N. Goteti and N. Kern, "Understanding Renewable Cost Projections and Planning Impacts: How Future Assumptions Shape U.S. Electric Sector Decarbonization Strategies,"

EPRI, Palo Alto, CA, 2021.

[15] J. Rutherford, E. Sherwin, A. Ravikumar, G. Heath, J. Englander, D. Cooley, D. Lyon, M. Omara, Q. Langfitt and A. Brandt, "Closing the methane gap in US oil and natural gas production emissions inventories," *Nature Communications,* vol. 12, no. 1, p. 1–12, 2021.

[16] N. Sepulveda, J. Jenkins, A. Edington, D. Mallapragada and R. Lester, "The Design Space for Long-Duration Energy Storage in Decarbonized Power Systems," *Nature Energy,* pp. 1-11, 2021.

[17] J. Bistline, "Metrics for Assessing the Economic Impacts of Power Sector Climate and Clean Electricity Policies," *Progress in Energy,* vol. 3, no. 4, p. 043001, 2021.

[18] E. Baik, K. Chawla, J. Jenkins, C. Kolster, N. Patankar, A. Olson, S. Benson and J. Long, "What Is Different About Different Net-Zero Carbon Electricity Systems?," *Energy and Climate Change,* p. Under Review, 2021.

[19] E. Larson, C. Greig, J. Jenkins, E. Mayfield, A. Pascale, C. Zhang, J. Drossman, R. Williams, S. Pacala and R. Socolow, "Net-Zero America: Potential Pathways, Infrastructure, and Impacts," Princeton University, Princeton, NJ, 2020.

[20] J. Bistline, "Roadmaps to Net-Zero Emissions Systems: Emerging Insights and Modeling Challenges," *Joule,* vol. 5, no. 10, pp. 2551-2563, 2021.

[21] J. Bistline and S. Rose, "Social Cost of Carbon Pricing of Power Sector CO2: Accounting for Leakage and Other Social Implications of Subnational Policies," *Environmental Research Letters,* vol. 13, no. 1, p. 014027, 2018.
